# Nutrients and soil structure influence furovirus infection of wheat

**DOI:** 10.3389/fpls.2023.1200674

**Published:** 2023-08-04

**Authors:** Kevin Gauthier, Dejana Pankovic, Miroslav Nikolic, Mirko Hobert, Christoph U. Germeier, Frank Ordon, Dragan Perovic, Annette Niehl

**Affiliations:** ^1^ Julius Kühn Institute (JKI) – Federal Research Centre for Cultivated Plants, Institute for Epidemiology and Pathogen Diagnostics, Brunswick, Germany; ^2^ Julius Kühn Institute (JKI) – Federal Research Centre for Cultivated Plants, Institute for Resistance Research and Stress Tolerance, Quedlinburg, Germany; ^3^ Institute for Multidisciplinary Research, University of Belgrade, Belgrade, Serbia; ^4^ State Institute for Agriculture and Horticulture Saxony-Anhalt, Centre for Agricultural Investigations, Bernburg, Germany; ^5^ Julius Kühn Institute (JKI) – Federal Research Centre for Cultivated Plants, Institute for Breeding Research on Agricultural Crops, Quedlinburg, Germany

**Keywords:** furoviru*s*, nutrients, *Polymyxa graminis*, wheat, modeling, prediction of infection rates, soil physical parameters, soil chemical parameters

## Abstract

*Soil-borne wheat mosaic virus* (SBWMV) and *Soil-borne cereal mosaic virus* (SBCMV), genus *Furovirus*, family *Virgaviridae*, cause significant crop losses in cereals. The viruses are transmitted by the soil-borne plasmodiophorid *Polymyxa graminis*. Inside *P. graminis* resting spores, the viruses persist in the soil for long time, which makes the disease difficult to combat. To open up novel possibilities for virus control, we explored the influence of physical and chemical soil properties on infection of wheat with SBWMV and SBCMV. Moreover, we investigated, whether infection rates are influenced by the nutritional state of the plants. Infection rates of susceptible wheat lines were correlated to soil structure parameters and nutrient contents in soil and plants. Our results show that SBWMV and SBCMV infection rates decrease the more water-impermeable the soil is and that virus transmission depends on pH. Moreover, we found that contents of several nutrients in the soil (e.g. phosphorous, magnesium, zinc) and *in planta* (e.g. nitrogen, carbon, boron, sulfur, calcium) affect SBWMV and SBCMV infection rates. The knowledge generated may help paving the way towards development of a microenvironment-adapted agriculture.

## Introduction

The availability of nutrients in the environment influences crop growth and thus impacts biomass, vitality, and grain quality or quantity of cereal crops (Chapin, 1980; Miao et al., 2006; Lee et al., 2017). It has been shown that the use of fertilizers allowed a yield increase of 50% during the 20^th^ century, or a yearly increase in crop milling quality of around 2% during 50 years (Fageria and Baligar, 2005; Gu et al., 2015). In addition to these direct effects on crops, nutrient availability acts as a major driver of interactions between organisms, shaping microorganism community composition and driving their interactions at both, the macroscopic (Borer et al., 2014) and microscopic scale, e.g. by regulating the abundance of bacteria (Francioli et al., 2016; Wang et al., 2018), fungi (Muneer et al., 2021) or viruses (Finke et al., 2017), as well as by modifying plant metabolism (Calabrese et al., 2017; Calabrese et al., 2019; Heyneke and Hoefgen, 2021). Carbon (C), nitrogen (N) and phosphorous (P) are believed to have a high impact on community composition according to the resource-ratio theory (Miller et al., 2005). Furthermore, the difference observed in C:N:P stoichiometry among virus, bacteria and fungi suggests that the different microorganisms have different needs for the different nutrients (Griffiths et al., 2012; Finke et al., 2017; Zhang and Elser, 2017). Reciprocally, living organisms directly influence the dynamics and availability of nutrients in their environment (Middelboe et al., 1996; Jones and Smith, 2004), especially in soils, in which they directly compete for nutrients. Beside its role as source of nutrients, soil has a physical role, serving as a support for plant roots, shaping their growth, driving water uptake (Jakobsen and Dexter, 1987) and influencing microbial activity (Jat et al., 2018). In return, roots and microorganisms influence the texture of soils, e.g. by storing organic matter or by colonizing the pores of the soil (Six et al., 2004). As pathogenic microorganisms cause substantial crop damage, studying the interactions between plants and pathogens is specifically important. However, it remains unclear how the availability of nutrients influences the plant- pathogen interaction. Besides their importance for plant growth by providing essential elements for the biosynthesis and function of proteins, nucleic acids, and sugars, macro- and micronutrients are involved in defense mechanisms against pathogens (Dordas, 2008). In the case of macronutrients, potassium (K) and nitrogen (N) were reported to promote a hypersensitivity response to infection as they are present in signaling molecules (Orober et al., 2002; Mur et al., 2017). Moreover, it has been shown that potassium increases the integrity of the cell wall and the speed of cell turgor recovery (Niehl et al., 2006; Wang et al., 2013). Concerning micronutrients, it has been reported that boron (B) favors cell wall integrity and improves the resistance to some pathogens (Dong et al., 2016), manganese (Mn) and copper (Cu) promote lignification (Eskandari et al., 2020), and zinc (Zn) is needed to form the zinc-finger domain in resistance proteins (Gupta et al., 2012). However, as nutrients play a role in pathogen growth and spread too, their role cannot be reduced to plant defense promoters, as their action seems to be very specific to each plant-pathogen-nutrient system. While some studies suggest that high nutrient availability increases the ability of plants to combat pathogens (Verly et al., 2020) or reduce the symptoms of infection (e.g. Chrpová et al., 2020; Chien and Huang, 2021), other studies report that it favors infection or leads to stronger disease symptoms (Snoeijers et al., 2000; Ballini et al., 2013).

Soil-borne pathogens may interact with soil nutrients directly or indirectly after infecting the host plant (DiTommaso and Aarssen, 1989). Soil-borne wheat mosaic virus (SBWMV) and Soil-borne cereal mosaic virus (SBCMV), genus *Furovirus*, family *Virgaviridae*, are among the most devastating cereal viruses worldwide, leading on average to yield losses of 30-40% (Adams, 1990; Lapierre and Hariri, 2008; Kroese et al., 2020). SBWMV and SBCMV are positive sense single stranded bi-partite RNA viruses (King et al., 2011; Niehl and Koenig, 2021). RNA1 encodes a replication protein, an RNA-dependent RNA polymerase and a movement protein while RNA2 encodes a major coat protein (CP) and a minor CP, initiated by an upstream start codon, a CP-RT protein produced from translational readthrough of the stop codon and a cysteine rich silencing suppressor protein (Te et al., 2005; Andika et al., 2016). Infections with SBWMV or SBCMV cause mosaic symptoms and light stunting, easily recognizable at field scale as chlorotic patches (Niehl and Koenig, 2021). The host range of SBWMV and SBCMV is relatively narrow, restricted to *Poaceae*, mostly cultivated, although infections of wild grasses of the genera *Bromus* were reported (McKinney, 1944; Lapierre and Signoret, 2004). The viruses are transmitted to the roots of cereals by the soil-borne plasmodiophorid *P. graminis.* Most of the infections of winter cereals are thought to occur in fall. The soil-borne motile spores of *P. graminis* reach the roots of the host plants by swimming in the free water of soils (Barr and Allan, 1982; Adams, 1990; Kanyuka et al., 2003). After reaching the roots, viruliferous *P. graminis* spores inject their cell content and along with it viruses into their host root cell. SBWMV and SBCMV replicate and move systemically in the host, while *P.graminis* replicates and locates only in the roots (Verchot et al., 2001). Viruliferous *P. graminis* spores are released from the roots into the soil where they can survive for decades (Rao and Brakke, 1969; Campbell, 1988), keeping contaminated soils infectious for a very long time.

*P. graminis* is currently divided into several formae speciales with different climatic preferences (Legrève et al., 2002). Currently, the only efficient disease control method involves breeding of resistant cultivars, relying on two known resistance genes in wheat: *Sbm1 and Sbm2* (Kanyuka et al., 2004; Bass et al., 2006; Perovic et al., 2009; Maccaferri et al., 2011). *Sbm1* and *Sbm2* are located on the 5DL and 2BS chromosomes, respectively (Bass et al., 2006; Liu et al., 2020). *Sbm1* and *Sbm2* were described as translocation resistances (Hunger and Sherwood, 1984; Driskel et al., 2002; Bass et al., 2006), not preventing the infection of roots, but limiting viral loads in the upper part of the plants to low amounts. The resistance mechanism underlying *Sbm3*, a recently identified additional resistance gene located on the A genome is still unknown (Schlegel et al., 2022). As the resources to combat SBWMV/SBCMV infection are very limited, optimizing the deployment of resistant plants and understanding additional factors influencing the infection is important.

In the SBWMV/SBCMV-*P. graminis*- host pathosystem it is well possible that the availability of nutrients in the soil and soil structure play a key role in determining the outcome of infection. *P. graminis* and the roots potentially compete for and modulate the uptake of micro- and macronutrients within the infected host. Nutrient availability within the host may determine the efficiency of virus replication and thus susceptibility of plants towards viruses. Soil structure and soil chemical composition likely influences the uptake of nutrients by the roots as well as the germination rate and movement of *P.graminis* spores. Here, we examined the influence of soil nutrient availability and uptake by plants, as well as soil structure on SBWMV and SBCMV infection rates of susceptible wheat lines in greenhouse conditions using infected soil collected from several naturally infested fields in France, Germany, Italy, and the England between 2017 and 2020. Both, single and interacting effects of the soil properties (pH, percentage of fine particles) nutrient contents in the soil and in plant roots and leaves on infection rates were analyzed using generalized linear models. Our results give new insight into the influence of soil condition and fertilization on SBWMV/SBCMV infection rates in wheat. This knowledge may help to develop a microenvironment-adapted agriculture.

## Materials and methods

### Soil collection

100-200 l of soil infected with SBCMV or SBWMV were collected from fields in France, Italy, England and Germany, respectively in the period from 2017 to 2020 (**Table S1**). Except for samples from Heddesheim (Ger) and England, soil samples from all other locations were collected in at least two subsequent years. For samples from England, soil from each field was collected twice. Before use the soil was kept at at 1°C to 6°C in the dark.

### Plant material

Three susceptible wheat lines, Avalon, Prevert and the durum wheat Pescadou were used. Seeds pre-germinated on filter paper at room temperature in the dark for three to five days were transplanted to pots (V= 520ml) filled with the infested field soils. Field soil were crushed to break up the larger clumps and were mixed before being filled into pots. Twenty plants were planted into each pot. Plants were grown in climate chambers at 14°C, 60% humidity, and a 16 h photoperiod with 10 klux. During the first 4 weeks plants were watered from the bottom and after that daily watering was applied. After 12 weeks in infected soil, the youngest leaves of each plant were harvested for the detection of SBCMV and SBWMV by ELISA. Subsequently, the foliage and roots of the plants were harvested for virus and *P. graminis* detection by RT-qPCR and for the analysis of nutrients. Roots were washed to remove soil particles and dried with filter paper to remove excess water. Leaf and root samples of six plants were pooled per wheat line, immediately snap frozen in liquid nitrogen and kept at -80°C.

### Element analysis

#### Determination of the grain size composition of soil

10g air-dried soil were suspended in 15% w/vol H_2_O_2_ for 15 h and then heated in a water bath at 90°C until organic matter was completely destroyed. 25 ml 0,4 N Na_4_P_2_O_7_ was added to the sample and incubated over night. The next day the sample volume was adjusted to 250 ml with distilled H_2_O and shaken for 6 h. Subsequently, the suspension was sieved through a 0.2 mm mesh into a 1000 ml glass cylinder and purged with distilled H_2_O to nearly 1000 ml. The grain size was determined by sieving through mesh sieves with 0,2 mm, 0,63 and 2,0 mm pore size. Resulting fractions for grain sizes were >0.2 mm, <0,2 to 0,63 mm <0,63 to 2,0 mm. The grain size fractions were dried at 105°C to constant weight. For the grain fraction <0.2 mm the glass cylinder was shaken six times overhead for 10 sec and then placed thermo-constant and vibration-free for sedimentation. For the individual fall-times of the fractions the pipette depth varied between 10 and 30 cm according to *DIN ISO 11277:2002-08* (Din, 2002). For every suspected fraction 10 ml suspension was pipetted to a scale dish and dried at 105°C to constant weight. The grain fractions were estimated in % weight in relation to dry matter.

#### Determination of pH, plant available phosphorous-, potassium- magnesium- and zinc contents and determination of nutrients in soil extracts

pH, plant available phosphorous-, potassium- and magnesium-contents were determined in soil extracts with the calcium-acetate-lactate (CAL) extraction method (Schüller, 1969). All other plant nutrients were determined in soil extracts according to VDLUFA Methodbook I (VDLUFA, 1991).

For determination of pH, plant available phosphorous and potassium, 5 g air-dried and to <2 mm sieved soil were suspended in 100 ml CAL-solution [0,05 M C_6_H_10_CaO_6_; 0,05 M (CH_3_COO)_2_Ca; 0,3 M acetic acid], agitated overhead for 90 min and then filtered through a MN 616 md ¼ filter (Macherey-Nagel, Düren, Germany). The first 20 ml of the filtrate was discarded. The following filtrates were analyzed with calibration standards for each parameter in bubble gating with a continuous-flow-analyzer (Skalar Analytical, The Netherlands) according to the manufacturer’s instructions. The pH sample was dialyzed against the indicator methyl red and photometric analysis performed at 540 nm. For plant-available phosphorous, extinction was measured photometrically at 880 nm and potassium was identified with a flame-photometer at 776 nm. Results were recorded in pH-Scale and mg per 100 g air-dried soil, respectively.

For identification of plant available magnesium, 5 g air-dried and to <2 mm sieved soil was suspended in 50 ml calcium chloride solution (0,01 M CaCl_2_), shaken for 120 min and then filtered through a MN 280 1/4 (Macherey-Nagel) filter. The first 15 ml of the filtrate were discarded. The remaining filtrate was analyzed with a continuous-flow-analyzer (Skalar Analytical) in a complex with xylidil blue according to the manufacturer’s instructions. The complex was measured photometrically in bubble gating at 470 nm with a calibration standard. Results were recorded in mg per 100 g air-dried soil.

For determination of plant available zinc 20 g air-dried and to <2 mm sieved soil was suspended in 100 ml EDTA-disodium salt-solution [0,05 M C_10_H_14_N_2_Na_2_O_8_], agitated overhead for 120 min and subsequently filtrated through a MN 616 md 1/4 (Macherey-Nagel) filter. The first 10 ml of the filtrate were discarded. The filtrate was analyzed with calibration standards with a ICP-OES 5100 VDV from Agilent (Agilent Technologies, Santa Clara, United States) according to manufacturer’s instructions. Results were recorded in mg per kg air-dried soil.

For analytical determination of total nitrogen 2 g air-dried and to <2 mm sieved soil was pulverized in a Pulverisette 5 (Fritsch, Idar-Oberstein, Germany) and analyzed with a Dumas-Analyzer (VarioMax, Elementar Analysesysteme, Langenselbold, Germany) with a calibration standard according to the manufacturer’s instructions. Results were recorded as % air-dried soil.

For all determined elements, the variation between two replicates of each analyzed soil sample was below 10%.

#### Determination of element content in plant tissue

Element contents of carbon (C) and nitrogen (N) were determined in root and shoot material with a Thermo EA1112 HT elemental analyzer according the Dumas combustion method. Samples were freeze dried at -20 °C, 1 mbar for 48 h in a CHRIST ALPHA 1-4 LD plus freeze-drier (Martin Christ GmbH, Germany). Freeze dried samples were ground to a fine powder using a ball mill and 2.200 µg weighed into tin capsules (3.3 x 5 mm, IVASA76980502). They were burned in a reactor with copper (IVASA99060102, IVA Analysentechnik, Meerbusch, Germany) as reductant, silvered cobaltous/IC oxide and chromium oxide (33824500 and 33822900, Thermo Fisher Scientific, Dreieich, Germany) as catalysts at 1020°C and resulting nitrogen (as N_2_) and carbon (as CO_2_) was determined by gas chromatography. Ratios of stable isotopes N^14^/N^15^, C^12^/C^13^ in these gases were determined in a Thermo Delta V Advantage isotope ratio mass spectrometer (IRMS). A certified wheat flour standard (IVA33802157: 1.36% N, 39.38% C, δ^15^N_air_ 2.85 ‰, δ^13^C_V-PDB_ -27.21 ‰) in amounts of 600, 1200, 1800 and 2400 µg was used for calibration, besides isotope calibration of reference gas N_2_ with standard materials from the International Atomic Energy Agency (IAEA N1, IAEA N2, USGS25). Each sample was measured in two runs.

For the analysis of phosphorous (P), potassium (K), magnesium (Mg), calcium (Ca), sulfur (S), manganese (Mn), copper (Cu), zinc (Zn), boron (B), and molybdenum (Mo), freeze dried plant material (0.2 g) was digested in 3 ml concentrated HNO_3 +_ 2 ml H_2_O_2_ for 1 h in a microwave oven (Speedwave MWS-3+; Berghof Products + Instruments GmbH, Eningen, Germany). The samples were diluted with deionised H_2_O (1:5) and subjected to multi-elemental analyses by ICP-OES (Spectro-Genesis EOP II, Spectro Analytical Instruments GmbH, Kleve, Germany). The certified reference material (GBW10015 Spinach; Institute for Geophysical and Geochemical Exploration, Langfang, China) was used to assess the accuracy and precision of the analyses.

#### Characterization of infection rates

After 12 weeks in infected soil, the youngest leaves of each plant were harvested for the detection of SBCMV and SBWMV by Double Antibody Sandwich Enzyme-Linked Immunosorbent Assay (DAS-ELISA) according Clark and Adams (1977), as explained in Schlegel et al. (2022). 96-well plates were coated with IgG 1:100 (JKI-AS92 for SBCMV and JKI-AS69 for SBWMV, respectively) in coating buffer (0.05M sodium carbonate, pH 9,6; 0.02% NaN_3_) for 4 h, washed four times with PBS pH 7,4 containing 0,05% Tween 20 using a Tecan ELISA washer (Tecan, Switzerland) and kept at -20°C until use. 50 mg fresh leaf or root material was homogenized (5000 rpm; 25 sec for leaf material and 50 sec for root at material) using metal beads in 500 µl of extraction buffer (PBS pH 7.4 containing 0.05% Tween 20, 2% polyvinylpyrrolidone MW 44,000, and 0.2% non-fat dry milk) using a Precellys homogenizer (Bertin Technologies, France). The extracts were incubated in coated ELISA plates overnight at 40 °C. The washing step consisted of four manual and four machine washing cycles (Tecan, Switzerland). The incubation step with the alkaline-phosphatase-labelled antibody (1:2000) was performed in a humid chamber at 37 °C for 4 h in conjugate buffer (PBS pH 7.4 containing 0.05% Tween 20, 2% polyvinylpyrrolidone MW 44,000, and 0.2% non-fat dry milk). After four washing cycles with a Tecan ELISA washer and incubation with 1 mg para-nitrophenylphosphate per ml substrate buffer (10% diethanolamine pH 9,8, 0.02% NaN_3_) for 1 h, the OD of 405 nm was measured (Sunrise, Tecan, Switzerland). Samples were considered positive when their measured OD exceeded two times the negative control with a minimum threshold of 0.1 after blank reduction. The infection rate was calculated as ratio of infected over total number of plants (%).

#### Determination of virus and *P. graminis* subspecies

##### RNA extraction

Snap frozen root samples were finely ground using a ball mill (Retch mill, MM301, Germany), precooled in liquid nitrogen and kept at -80 °C. RNA extraction was performed with the ground root samples using NucleoZol (Macherey-Nagel, Düren, Germany) reagent according to the manufacturer’s instructions. Briefly, 0.05 g of ground material were suspended in the same volume of reagent. After vortexing and centrifugation, RNA was precipitated using isopropanol and washed twice with ethanol before solubilization.

##### RT real-time PCR

Virus species and vector sub-species, respectively were identified with reverse transcription real time PCR (RT-qPCR) and reverse transcription real time nested PCR (RT-nested qPCR). Reverse transcription was performed on the extracted RNA after 10-fold dilution. 1 μL of the diluted RNA, 10 μmol of random primers, 2μL DTT and 20U ProtoScript^®^II Reverse Transcriptase (New England Biolabs, Ipswich, United States), 125 μmol of each dNTP and 4μL provided buffer were used for the synthesis according to manufacturer’s instruction with minor modifications in a 20 μL reaction. Samples were placed in a FlexCycler PCR System (Analytik Jena, Jena, Germany) with the following cycle parameters: 25 °C for 5 min, reverse transcription at 42 °C for 1 hour followed by a step of 20 min at 65 °C for inactivation. For *Polymyxa* identification, the produced cDNA (of ITS-derived sequences specific for *P. graminis ssp. tepida* and *ssp. temperata*, respectively) were amplified using 2 μL cDNA, 500 nmol of each primer (PGG_166_FW and PGG_631_RV, **Table S2**), 125 μmol of each dNTP and 4 μL of buffer provided with 5 U One *Taq* polymerase (New England Biolabs) according to manufacturer’s instructions with minor modifications in a 20 μL reaction. Samples were placed in a FlexCycler with the following cycle parameters: 5 min at 94 °C followed by 15 cycles of 30 seconds at 94 °C, 1min at 50 °C and 2 min at 68 °C concluded with 10 min at 72 °C for final extension. The amplified PCR products were 10-fold diluted to serve as template for real-time PCR. Real-time PCR for the detection of *P. graminis* subspecies after PCR or for detection of SBWMV or SBCMV after cDNA synthesis was performed using a qTower 2.2 (Analytik Jena) with 1 μL template, 500 nmol of each primer (1-BWF-6541T and 1-BWR-6683 for SBWMV, PTP-409-FW and PTP-512-RV for *P.graminis ssp. tepida*, PTM-403-FW and PTM-481-RV for *Polymyxa graminis ssp. temperata*, **Table S2**), and Luna^®^ Universal qPCR Master Mix (New England Biolabs) in a 10 μL reaction volume. The obtained Ct-values and melting temperatures were compared to standard curves and considered as positive when the measured melting temperature of the amplified product was less different than 1 °C when compared with the melting temperature of the highest concentration present in the melting curve and the average number of cycles needed for detection was lower than 35.

### Preparation of the standard curves

After RNA extraction (as described above) a step of reverse transcription was performed (as described above). The cDNAs were amplified as described above but with the primers 1-BWF-6237 and 1-BWR-7018 for SBWMV, PGG-51-FW and PGG-882-RV for *P. graminis.* After a 10-fold dilution of the amplified DNA for *P. graminis*, another amplification was performed in the same conditions with the primers PGG_166_FW and PGG_631_RV. Amplified DNA was visualized and cut from Midori green stained gels and purified using a Nucleo Spin^®^ PCR and Gel kit (Macherey-Nagel). Purified products were inserted into pDrive Cloning vector using a PCR Cloning Kit (QIAGEN, Venlo, Netherlands). Next, electro competent *Escherichia coli* of the DH5α strain were transformed using an Electroporator ^®^ (Eppendorf, Hamburg, Germany). Concisely, bacteria were shortly incubated on ice before electroporation at 2500 V. After 1 h regeneration time in LB at 37 °C, 700 rpm in a Thermomixer (Eppendorf), cells were plated on agar plates containing 50 ng carbenicillin before being cultivated overnight at 37 °C. The presence of the insert in colonies was analyzed using the same primers as used for the cloning. Colonies containing the inserts of interest were cultivated in liquid agar at 37 °C over night. Plasmids were finally extracted using a Nucleo Spin^®^ Plasmid kit (Macherey-Nagel) and sequenced (Eurofins MWG, Ebersberg, Germany) to confirm the identity of the insert.

### Statistical analysis

All statistical analyses were performed using R version 3.6.3 (R Development Core Team, 2019). All the variables were analyzed using principal component analysis (PCA) from the package FactoMineR (Lê et al., 2008). All data were normalized before analysis by normalizing each value to the mean of all samples for the respective measured parameter divided by SD. Three modeling approaches were used to determine the influence of the studied parameters for the SBWMV/SBCMV infection rates. In the first approach, parameters influencing the infection rates in the soils were selected after a stepwise backward modelization (STBM) (Whittingham et al., 2006) in binomial or quasi-binomial generalized linear models, based on all studied parameters for soil parameters and plant nutrients. Every parameter was analyzed for its significance in the model and the parameter with the highest non-significant p-value was removed from the model. The model was re-evaluated until every remaining parameter was significant or no parameter remained in the model.

This modelization procedure was repeated for the element analysis performed with plant samples. A final Holm correction was performed on the obtained p-values (Holm, 1979). After identification of the parameters influencing the infection rates, predictive generalized linear models were used to determinate their exact influence on the SBWMV and SBCMV infection rates. In the second approach, inspired from Lacroix and collaborators (2017), the function dredge from the R package MuMin was used to fit all the possible models. A prior step of stepwise backward selection was eventually performed in case that the number of factors and interactions exceeded the number of analyzed samples. Parameter values, errors and AIC [Akaike information criterion, or QAIC (quasi Akaike information criterion)], respectively were estimated using the function model.avg using all the models with a delta value smaller than 4 (MAVG). The third approach used was a mixed approach between the first and the second method, in which the selected parameters after model averaging were checked (ST-MA) for their significance before being kept in the model. The significance of the tested factors was assessed using Fisher tests for STBM methods and Walt tests for the two other approaches. In the case of ST-MA, the weight of each parameter was determined between zero (irrelevant parameter) and one (parameter present in every model). Infection rates were predicted according to the identified factors using the function predict.glm. The predicting performance of STBM and ST-MA were then compared. For representation, in case the model contained more than four significant factors, only the four first are presented. Finally, a hierarchical classification of the soils relying on the tested parameters was performed by using the package dendextend (Galili, 2015). The number of groups was chosen according to the highest drop of inertia. All the graphics were drawn with the ggplot (Hadley, 2016), sjPlot and stats packages. Original data for all physical and chemical soil and plant parameters measured are presented in **Supplemental Table S3**.

## Results

### Soil classification and element analysis

**Figure 1:**
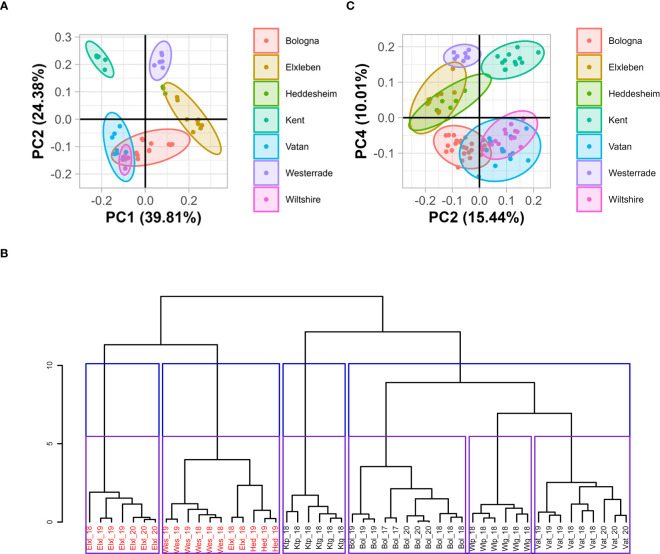
Presentation of the dataset obtained for soil data using PCA and hierarchical clustering **(A, B)** and of the dataset obtained for plant data using PCA **(C)**. **(A)** PCA on soil properties of the seven locations with seven parameters analyzed after normalization. Data were normalized to the mean of all samples for the respective measured parameter and divided by SD. 95% confidence intervals are represented by ellipses. No ellipse was drawn for Heddesheim data, as only three sampls were available for this location. **(B)** Hierarchical classification by ascendance of the studied soils based on their physio-chemical properties. Bol: Bologna, Elxl: Elxleben, Hed: Heddesheim, Ktg: used Kent, Ktp: new Kent, Vat: Vatan, Wes: Westerrade, Wlg: used Wiltshire, Wltp: new Wiltshire. The number indicates the year of soil sampling. The ordinate shows the inertia of the analysis. Soils containing SBWMV are displayed in red, soils containing SBCMV are displayed in black. Blue and purple squares display the clustering in four or six groups, respectively as suggested by the analysis of the inertia drop (**Figure S4**). Three replicates of each soil per year were analyzed with the exception of Bol_17, where two replicates were analyzed. **(C)** Principal component analysis on element composition of leaf and root samples of plants grown in viruliferous *P. graminis*-containing soil using the second and fourth principal component. Samples were taken from plants grown in soils from the different fileds, respectively for 12 weeks. Thirteen parameters were analyzed after normalization (**Figure S6**). 95% confidence intervals are represented by ellipses.

To gain a first overview about the differences between the soils used in our study, we conduced principal component analysis (PCA) on the measured soil parameters (**Figure 1A** and **Supplemental text**, **Figure S1**). All the studied soil parameters (percentage of fine particles, pH, available phosphorus (PCAL extractable) and potassium (KCAL extractable) quantities of plant available magnesium (Mg), nitrogen and zinc (Zn) had a similar contribution to the PCA and allowed a clear separation of the tested soils according to their geographical location (**Figure 1A**). Component 1 (PC1), explaining 39.8% of the variance of soil properties (**Figure S1**), was mostly associated with magnesium content, plant-available phosphorus (PCAL), plant-available potassium (KCAL) and pH. Samples with high Eigenvector values in PC1 were associated with high PCAL and alkaline pH but with low magnesium content and low KCAL. The percentage of fine particles accounts for almost 50% of the observed variance in the component 2 (PC2), which explains 24.4% of the variation in the dataset. Component 3 (PC3) displays the difference in nitrogen and, to a lesser extent, zinc content. Soils with high Eigenvector values in PC3 have high nitrogen and zinc contents (**Figures S2**, **S3**). We also classified the data by hierarchical ascendance using the studied parameters (**Figures 1B**, **S4**). With the exception of two samples gathered in Elxleben in 2018, all samples derived from the same location clustered in the same group (**Figures 1B**, **S4**).

When PCA was performed with nutrition-associated parameters measured in leaf and root samples grown in the different soils, the highest variance was between the leaves and the roots (**Figures S5**, **S6**). The segregation of the dataset into leaves and roots was mainly described by δ^13^C, nitrogen and potassium contents, as these parameters have the highest influence on PC1 (**Figures S7**, **S8**). Interestingly, when PC2 was plotted against PC4, the dataset was separated into the different locations from which the samples were derived (**Figure 1C**). PC2 explains 15.44% of the variance, and the coordinates in PC2 represented high calcium, sulfur and zinc contents and low δ^15^N. Low molybdenum quantities and high δ^13^C as well as carbon content values constituted high Eigenvector values in PC4 (**Figures S7**, **S8**).

### Identification of vectors and viruses in relation to the soils and their physico-chemical properties

**Figure 2:**
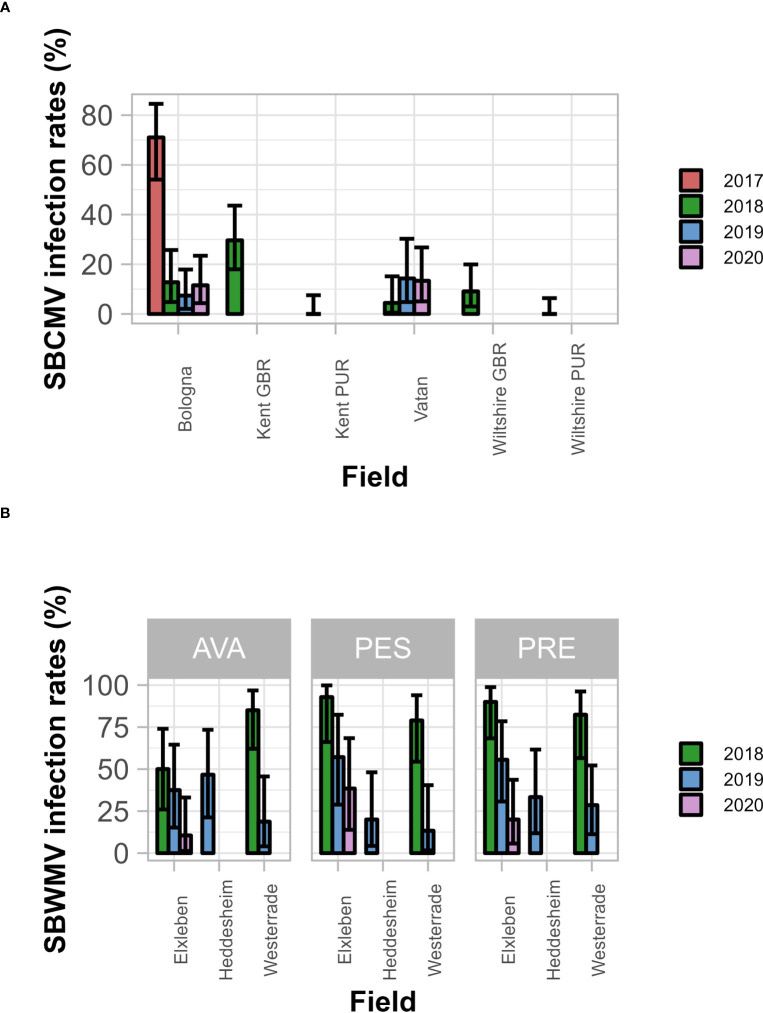
SBCMV **(A)** and SBWMV **(B)** infection rates of the different cultivars. Only the significant parameters for each virus are presented. Error bars represent 95% confidence intervals. A generalized linear mixed effects model was applied and significance determined *via* ANOVA. **(A)**, infection rates differ between soils (p=2.75E-4) and years (p=2.90E-5); not between the cultivars (p=0.45); **(B)**, infection rates differ between soil – year interaction (p=0.0096) and between soil - cultivar interaction (p=0.014). AVA, Avalon; PES, Pescadou; PRE, Prevert. Error bars represent 95% confidence intervals.

Presence of SBCMV and SBWMV in plants grown in different soils was analyzed by RT-qPCR. Consistent with previous findings (Kastirr and Ziegler, 2018), we found SBCMV in plants grown in soil from Bologna, Kent, Vatan and Wiltshire and SBWMV in plants grown in the soil from Elxleben, Heddesheim and Westerrade (**Tables S4A, B**). No co-infection with the respective other virus was observed. *P. graminis ssp. tepida* was identified in the roots of plants grown in soil form each location. By contrast, *P. graminis ssp. temperata* was only identified in the roots of plants grown in Elxleben, Kent and Vatan (**Table S4B**). Co-infections *with P. graminis ssp. tepida* and *temperata* occurred in Vatan (*Avalon*, 2020) and in Kent (*Prevert* and *durum Pescadou*, 2018). Interestingly, hierarchical ascendance clustering revealed a clear separation of soils in which SBWMV and soils in which SBCMV was present (**Figure 2**, red and black writing), demonstrating that SBWMV and SBCMV require different soil parameters. The classification by hierarchical ascendance did not highlight any relation between soil properties and *P. graminis* subspecies. No relation between virus species and vector subspecies was established either.

### Analysis of infection rates

SBCMV infection rates ranged from 0% to 88.2% (**Figure 2A**), and SBWMV infection rates ranged from 10.2% to 92.6% (**Figure 2B**). Thus, we concluded that the data set contained enough variability to investigate the influence of soil- and nutrition parameters on infection efficiency. The factors field (p=2.75E^-4^) and year (p=2.90E^-5^) significantly affected SBCMV infection rates, while the cultivar did not (p=0.45). SBWMV infection rates were influenced by the interactions between year and field factors (p=9.60E^-3^) and between cultivar and field (p=0.014).

To investigate the influence of soil properties on SBCMV and SBWMV infection rates and predict the influence of specific parameters on virus infection rates, different modeling approaches were applied and compared. We used STBM as a simple means to identify the most significant parameters and compared it with the results obtained by using MAVG. MAVG reveals all possible relevant contributions and finds the best model to describe the data, according to AIC or QAIC. However, MAVG is prone to generate underdispersed models by maintaining non-significant parameters, especially when the number of tested parameters is high. Thus, we combined STBM and MAVG approaches and applied a stepwise model averaging (ST-MA) approach.

Concerning the influence of soil proprieties on SBCMV infection rates, STBM identified only the interaction between soil pH and the percentage of fine particles as relevant factors to explain the observed differences in SBCMV infection rates (p=0.023, **Table 1**). By contrast, MAVG did not identify any significant parameter. The ST-MA analysis identified the amount of fine particles (p=1.94E^-7^), magnesium (p=1.61E^-11^), PCAL (p=0.021) and pH (p=1.19E^-11^) as parameters to explain the observed differences in SBCMV infection rates (**Table 1**). PCAL, however, weighed less in the ST-MA model compared to the other factors (relative importance of 0.15 against 1 for all other factors).

**Table 1:** Summary of the effects of soil parameters on SBCMV infection rates according to the three tested models.

Model	STBM			MAVG			ST-MA			
Factor	Value	Error	sg	Value	Error	sg	Value	Error	sg	Weight
Intercept	-138.8	57.16	0.02^*^	-4675.4	2663983.1	0.999	-26.981	5.265	2.98E-07^***^	NT
pH	21.13	8.56	0.325	449.0	360259.0	0.999	4.146	0.611	1.19E-11^***^	1
FP	3.45	1.58	0.0625	191.4	16458.8	0.991	-0.240	0.046	1.94E-07^***^	1
pH x FP	-0.53	0.24	0.023^*^	-20.1	2453.1	0.993	NT	NT	NT	NT
Mg	NT	NT	NT	36.5	69084.1	1.000	0.363	0.054	1.61E-11^***^	1
PCAL	NT	NT	NT	-226.4	122025.1	0.999	0.140	0.061	0.021^*^	0.15
FP x Mg	NT	NT	NT	-1.1	226.2	0.996	NT	NT	NT	NT
FP x PCAL	NT	NT	NT	-5.5	595.7	0.993	NT	NT	NT	NT
Mg x pH	NT	NT	NT	-8.0	7096.3	0.999	NT	NT	NT	NT
PCAL x pH	NT	NT	NT	60.5	18706.7	0.997	NT	NT	NT	NT
Mg x PCAL	NT	NT	NT	6.7	845.2	0.994	NT	NT	NT	NT

sg, significance; NT, not tested (the parameter was not included in the respective model); intercept, intercept of the model; FP, Amount of fine particles (%); PCAL, Calcium acetate lactate extractable phosphate (mg*100g^-1^ dry soil); Mg, plant-available magnesium (mg*100g^-1^ dry soil). Significance of effects is indicated by a 0.05 (*) and 0.001 (***) threshold.

SBWMV infection rates turned out to be influenced by the interaction between the amount of zinc and the percentage of fine particles (p=0.01), as well as by PCAL (p=5.6E^-3^), when the STBM procedure was used (**Table 2**). MAVG identified the interaction between the quantity of fine particles in the soil and PCAL (p=0.024) and the interaction between zinc content and PCAL (p=1.3E^-4^) as factors influencing SBWMV infection rates and ST-MA resulted in the same predictions for SBWMV infection rates compared to the MAVG procedure.

**Table 2:** Summary of the effects of soil parameters on SBWMV infection rates according to the three tested models.

Model	STBM			MAVG			ST-MA			
Factor	Value	Error	sg	Value	Error	sg	Value	Error	sg	Weight
Intercept	71.34	20.57	0.004^**^	-123.46	68.03	0.070	-123.46	68.03	0.070	NT
Zn	-13.88	4.94	0.167	-2.31	14.93	0.877	-2.31	14.93	0.877	1
FP	-2.25	0.72	0.298	4.37	2.63	0.097	4.37	2.63	0.097	1
PCAL	-0.96	0.33	5.6E-3^**^	29.43	4.90	1.93E-09^***^	29.43	4.90	1.93E-09^***^	1
Zn x FP	0.47	0.17	0.01^**^	NT	NT	NT	NT	NT	NT	1
PCAL x FP	NT	NT	NT	-0.99	0.44	0.024^*^	-0.99	0.44	0.024^*^	0.83
Zn x PCAL	NT	NT	NT	-1.70	0.45	0.00013^***^	-1.70	0.45	0.00013^***^	1

sg, significance; NT, not tested (the parameter was not included in the respective model); intercept, intercept of the model; FP, Amount of fine particles (%); PCAL, Calcium acetate lactate extractable phosphate (mg*100g^-1^ dry soil); Zn, plant-available zinc content (mg*kg^1^ dry soil). Significance of effects is indicated by a 0.05 (*), 0.01 (**), and 0.001 (***) threshold.

We also applied the modeling approaches to the plant samples to investigate if the concentration of specific nutrients inside plant roots and leaves may influence infection. For SBCMV, STBM identified δ^15^N as factor to explain the observed differences in SBCMV infection rates in leaves (p=1.87E^-4^) and roots (p=3.04E^-5^) (**Tables 3**, **4**). MAVG identified the interaction between δ^15^N and calcium content (p=0.011), as well as the interaction between calcium and sulfur contents (p=0.009) and between boron and potassium (p=0.027) to influence SBCMV infection rates in leaves. For roots, MAVG predicted an influence of copper (p=0.038) and the interaction between boron and calcium (p=0.049) contents on SBCMV infection rates. ST-MA predicted the interaction between calcium and the sulfur quantities (p=1.78E^-4^), boron (p=5.29E^-8^) and copper (p=0,011) content, as well as δ^15^N (p=6.70E^-6^) to influence SBCMV infection rates in leaves (**Table 3**). For roots, ST-MA identified the interaction of calcium with boron (p=3.58E^-4^) and sulfur with boron (p=0.011), interaction between calcium and sulfur (p=2.0E^-3^), interaction between calcium and δ^15^N (p=1.00E^-3^), and interaction between copper and potassium (p=3.00E^-3^) influencing SBCMV infection rates (**Table 4**).

**Table 3:** Summary of the effects of nutrients measured in leaves on SBCMV infection rates according to the three tested models.

Model	STBM			MAVG			ST-MA			
Factor	Value	Error	sg	Value	Error	sg	Value	Error	sg	Weight
Intercept	2.08	0.88	0.02^*^	*3.42*	21.4	0.872	6.232	1.758	3.94E-04^***^	1
δ^15^N	-0.51	0.12	1.87E-04^***^	1.33	1.72	0.439	-0.513	0.114	6.70E-06^***^	1
B	NT	NT	NT	3.86	2.05	0.060	0.208	0.038	5.29E-08^***^	1
Cu	NT	NT	NT	-1.76	2.48	0.476	-0.168	0.066	0.011^*^	1
S	NT	NT	NT	-8.49	30.11	0.778	-25.884	7.059	2.45E-04^***^	1
Ca	NT	NT	NT	-64.95	27.62	0.019^*^	-4.705	1.447	1.14E-03^***^	1
K	NT	NT	NT	8.56	9.84	0.384	NT	NT	NT	NT
B x Cu	NT	NT	NT	-0.02	0.04	0.502	NT	NT	NT	NT
Cu x S	NT	NT	NT	-6.71	4.09	0.101	NT	NT	NT	NT
B x S	NT	NT	NT	0.06	1.69	0.974	NT	NT	NT	NT
Cu x δ^15^N	NT	NT	NT	-0.16	0.38	0.672	NT	NT	NT	NT
B x δ^15^N	NT	NT	NT	-0.31	0.16	0.056	NT	NT	NT	NT
δ^15^N x Ca	NT	NT	NT	5.29	2.1	0.011^*^	NT	NT	NT	NT
Cu x Ca	NT	NT	NT	6.44	4.59	0.161	NT	NT	NT	NT
δ^15^N x K	NT	NT	NT	-1.31	0.84	0.119	NT	NT	NT	NT
B x Ca	NT	NT	NT	-3.22	2.02	0.110	NT	NT	NT	NT
S x Ca	NT	NT	NT	60.71	23.53	0.009^**^	24.327	6.487	1.78E-04^***^	1
B x K	NT	NT	NT	0.83	0.38	0.027^*^	NT	NT	NT	NT
Cu x K	NT	NT	NT	-0.70	1.03	0.497	NT	NT	NT	NT
S x K	NT	NT	NT	-0.98	16.20	0.952	NT	NT	NT	NT

sg, significance; NT, not tested (the parameter was not included in the respective model); intercept, intercept of the model. δ^15^N, ^15^N/^14^N isotopic ratio in the leaves; B, boron content in the leaves (ppm); Cu, copper content in the leaves (ppm); S, sulfur content in the leaves (%); Ca, calcium content in the leaves (%); K, potassium content in the leaves (%). Significance of the effects is indicated by a 0.05 (*), 0.01 (**), and 0.001 (***) threshold.

**Table 4:** Summary of the effects of nutrients measured in roots on SBCMV infection rates according to the three tested models.

Model	STBM			MAVG			ST-MA			
Factor	Value	Error	sg	Value	Error	sg	Value	Error	sg	Weight
Intercept	2.57	0.85	0.005^**^	-66.63	32.06	0.038^*^	-39.219	9.983	8.55E-05^***^	1
δ^15^N	-0.63	0.13	3.04E-05^***^	2.740	2.376	0.249	1.731	0.522	9.09E-04^***^	1
B	NT	NT	NT	0.785	2.181	0.718	0.394	0.427	0.355	1
Cu	NT	NT	NT	0.825	0.398	0.038^*^	0.600	0.187	1.37E-03^**^	1
S	NT	NT	NT	192.0	110.5	0.082	104.977	29.773	4.22E-04^***^	1
Ca	NT	NT	NT	66.97	50.86	0.188	15.571	6.661	0.019^*^	1
K	NT	NT	NT	2.635	3.323	0.937	12.414	4.541	0.006^**^	1
B x Cu	NT	NT	NT	-0.012	0.027	0.641	NT	NT	NT	NT
Cu x S	NT	NT	NT	-1.570	0,983	0.873	NT	NT	NT	NT
B x S	NT	NT	NT	-21.74	13.39	0.105	-9.929	3.882	0.011^*^	1
Cu x δ^15^N	NT	NT	NT	8.3 E-04	0.016	0.958	NT	NT	NT	NT
B x δ^15^N	NT	NT	NT	0.219	0.215	0.308	NT	NT	NT	NT
δ^15^N x Ca	NT	NT	NT	-8.58	5.874	0.144	-2.674	0.813	0.001^**^	1
Cu x Ca	NT	NT	NT	-0.314	0.237	0.184	NT	NT	NT	NT
δ^15^N x K	NT	NT	NT	1.610	3.532	0.649	NT	NT	NT	NT
B x Ca	NT	NT	NT	5.276	2.683	0.049^*^	2.287	0.641	3.58E-04^***^	1
S x Ca	NT	NT	NT	-140.4	104.7	0.180	-57.224	18.36	0.002^**^	1
B x K	NT	NT	NT	-1.495	1.119	0.181	NT	NT	NT	NT
Cu x K	NT	NT	NT	-0.481	0.254	0.058	-0520	0.175	0.0030^**^	1
S x K	NT	NT	NT	30.26	94.48	0.749	NT	NT	NT	NT

sg, significance; NT, not tested (the parameter was not included in the respective model); intercept, intercept of the model δ^15^N, ^15^N/^14^N isotopic ratio in the roots; B, boron content in the roots (ppm); Cu, copper content in the roots (ppm); S, sulfur content in the roots (%); Ca, calcium content in the roots (%); K, potassium content in the roots (%). Significance of the effects is indicated by a 0.05 (*), 0.01 (**), and 0.001 (***) threshold.

For leaves, concerning SBWMV, STBM identified the interaction between carbon and manganese content (p=5.2E^-4^) along with δ^13^C (p=0.033) as the only significant factors explaining SBWMV infection rates variation (**Table 5**). MAVG further identified interactions between δ^13^C and carbon content (p=1.45E^-4^) between δ^13^C and manganese content (p=0.014) to influence SBWMV infection rates. Interaction between carbon and nitrogen content was also shown to have an influence (p=5.75E^-4^). ST-MA identified the interaction between the carbon and manganese content (p=3.00E^-3^) as influencing the SBWMV infection rates while the δ^13^C lost its significance upon averaging (p=0.065). δ^13^C was however represented in 85% of the tested models. For roots, all three modeling approaches (STBM, MAVG and ST-MA) failed to identify any relevant effect linked to SBWMV infection rates.

**Table 5:** Summary of the effects of nutrients measured in leaves on SBWMV infection rates according to the three tested models.

Model	STBM			MAVG			ST-MA			
Factor	Value	Error	sg	Value	Error	sg	Value	Error	sg	Weight
Intercept	-31.5	8.470	2E-04^**^	-925.2	267.1	5.32E-04^***^	-29.57	9.015	0.036^*^	1
C% dm	0.59	0.183	1.2E-03^**^	25.39	6.843	2.07E-04^**^	0.594	0.183	0.005^**^	1
Mn	0.50	0.150	8,1 E-04^***^	-0.692	0.510	0.175	0.492	0.150	0.004^**^	1
δ^13^C	-0.30	0.142	0.033^*^	-35.87	9.544	1.71E-04^***^	-0.302	0.142	0.065	0.79
C% dm x Mn	-0.01	0.004	5,2 E-04^***^	-0.008	0.009	0.354	-0.013	0.004	0.003^**^	1
N%	NT	NT	NT	-202.7	92.93	0.029^*^	NT	NT	NT	NT
C% dm x N% dm	NT	NT	NT	2.784	0.808	5.75E-04^***^	NT	NT	NT	NT
C% dm x δ^13^C	NT	NT	NT	0.959	0.252	1.45E-04^***^	NT	NT	NT	NT
δ^13^C x Mn	NT	NT	NT	0.038	0.015	0.014^*^	NT	NT	NT	NT
N% dm x δ^13^C	NT	NT	NT	-2.724	2.253	0.226	NT	NT	NT	NT
N% dm x Mn	NT	NT	NT	0.234	1.861	0.208	NT	NT	NT	NT

sg, significance; NT, not tested (the parameter was not included in the respective model); intercept, intercept of the model. C% dm, carbon content in the leaves (% dry matter (dm)); Mn, manganese content in the leaves (ppm); δ13C, ^13^C/^12^C isotope ratio in the leaves; N% dm, nitrogen content in the leaves (% dry matter (dm)). Significance of the effects is indicated by a 0.05 (*), 0.01 (**), and 0.001 (***) threshold.

### Prediction of SBCMV and SBWMV infection rates based on the factors identified by the different algorithms

**Figure 3:**
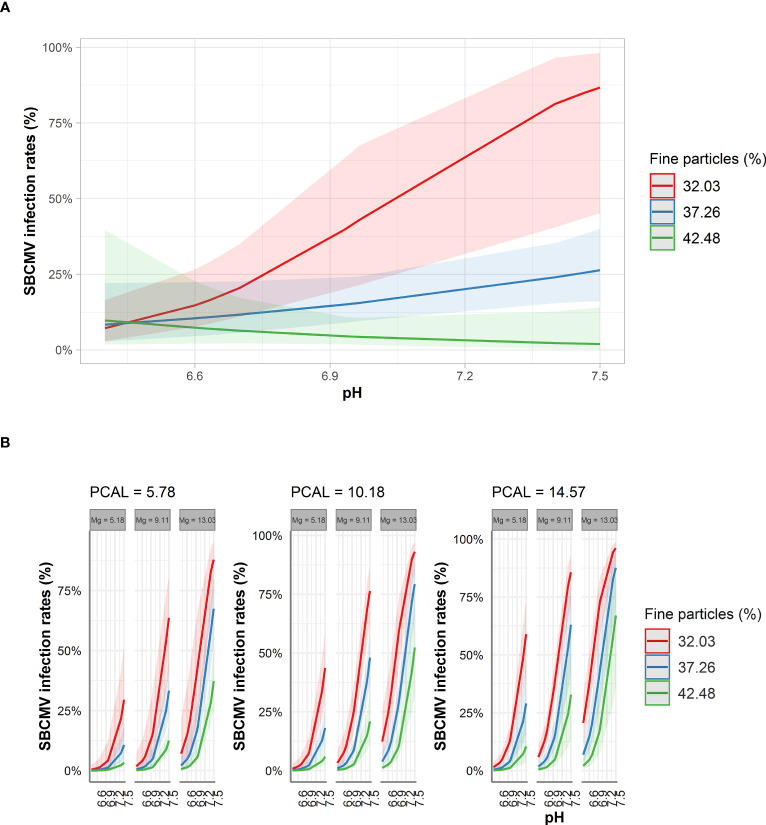
Predicted SBCMV infection rates based on soil parameters identified by STBM **(A)** and ST-MA **(B)**. Only the significant parameters for each model are presented. The colored zones depict 95% confidence intervals. Mg: plant-available magnesium (mg*100g^-1^ dry soil), PCAL: Calcium acetate lactate extractable phosphate (mg*100g^-1^ dry soil).

For the prediction analysis, we chose STBM and ST-MA procedures, as these procedures led to the identification of the most crucial soil and nutrient parameters influencing infection rates without the risk of keeping non-significant factors. Using the factors identified by the element analysis of soil, STBM predicted SBCMV infection rates to increase from 5% to 87% for a percentage of fine particles in the soil of 32.03% when pH increased from slightly acidic to slightly alkaline (6.45 to 7.5). For the same pH values, infection rates were predicted to increase from 5% to 25% for 37.26% of fine particles. In contrast, for 42.48% fine particles, the percentage of infection was predicted to decrease from 5% to 0% when pH Increased from 6.5 to 7.5 (**Figure 3A**) Thus, according to the STBM model, an decreasing amount of fine particles, coupled with an increasing pH from slightly acidic to slightly alkaline increases SBCMV infection rates. Consistent with our results from STBM, ST-MA predicted an increase of SBCMV infection rates when pH increased from 6.45 to 7.5. This increase in SBCMV infection rates was higher, when more magnesium was present in the soil. In addition, increasing PCAL increased infection rates (**Figure 3B**). Moreover, a lower fine particle content was predicted to increase SBCMV infection compared to higher content of fine particles (**Figure 3B**).

**Figure 4:**
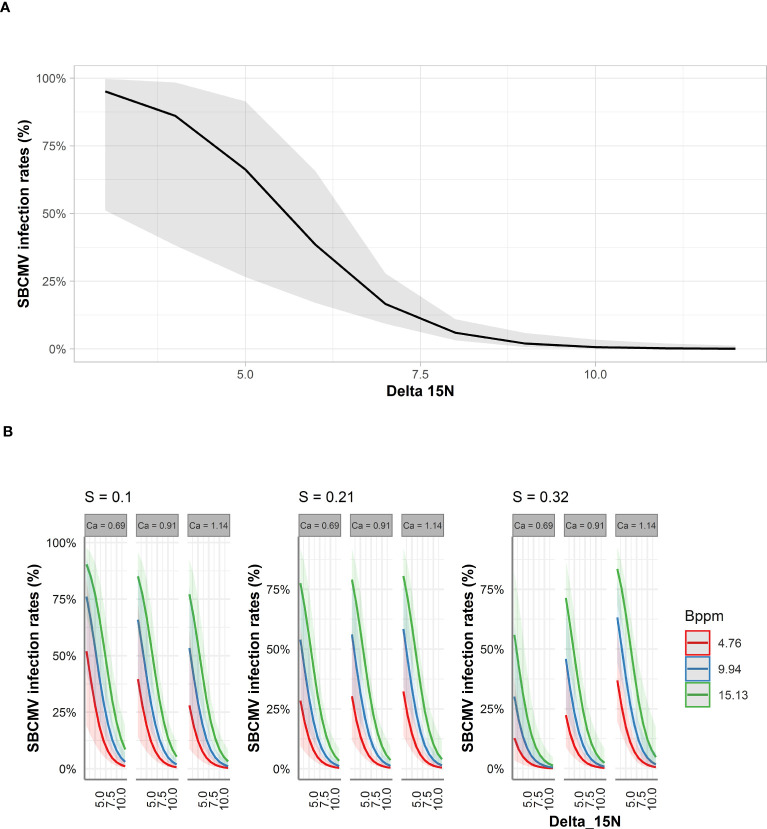
Predicted SBCMV infection rates based on STBM **(A)** and ST-MA **(B)** modeling of element data measured in leaves. Only the significant parameters for each model are presented. The colored zones depict 95% confidence intervals. Delta_15N: ^15^N/^14^N isotope ratio in the leaves, S: % sulfur content in the leaves, Ca: % calcium content in the leaves, Bppm: boron (ppm) content in the leaves.

**Figure 5:**
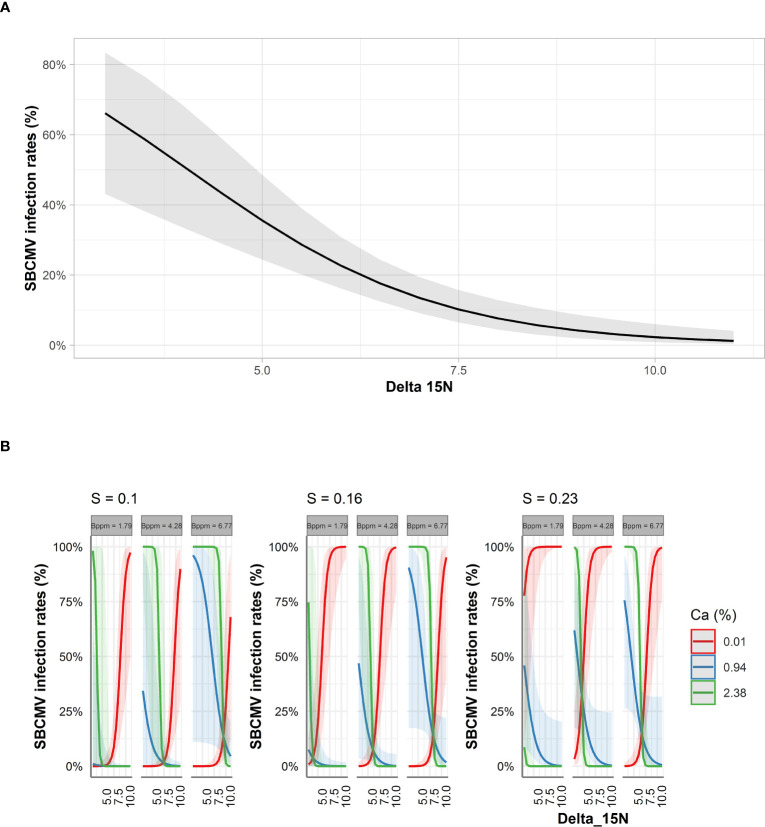
Predicted SBCMV infection rates based on STBM **(A)** and ST-MA **(B)** modeling of element data measured in roots. Only the significant parameters for each model are presented and only the four most represented parameters are shown in the figure. The colored zones depict 95% confidence intervals. Delta_15N: ^15^N/^14^N isotope ratio in the roots, S: % sulfur content in the roots, Bppm: boron (ppm) content in the roots, Ca: % calcium content in the roots.

For leaves and roots we predicted that δ15N decreased SBCMV infection rates about 90% in the leaves and 60% in the roots according to STBM (**Figures 4A**, **5A**). When ST-MA was used to model the effect of elements in leaves, decreasing δ^15^N levels also led to increasing SBCMV infection rates (**Figure 4B**). Moreover, a boron content augmentation from 4.7 to 15.1 ppm is predicted to increase SBCMV infection rates by 30%. Calcium and sulfur in leaves were predicted to synergistically affect SBCMV infection rates in leaves. SBCMV infection rates were high when calcium and sulfur were low or when both were high. When one of the two parameters was high and the other low, infection rates dropped (**Figure 4B**). ST-MA further predicted a 10% increase of SBCMV infection rates when copper content of leaves decreased from 12.67 to 4.70 ppm (result not displayed).

Among the factors predicted by ST-MA to influence infection rates in roots, was an increase of the SBCMV infection rates when δ^15^N decreased and calcium content increased (**Figure 5B**). ST-MA further predicted an up to 60% increase in SBCMV infection rates when boron quantity increased (from 1.7 ppm to 6.7 ppm). This effect was most prominent when sulfur quantities were low. By contrast, SBCMV infection rates were predicted to increase up to 75% when sulfur quantity decreased (from 0.23% to 0.10%); this effect was more pronounced when boron quantities were low and calcium quantities high. However, ST-MA also predicted that the effects of δ^15^N, boron and sulfur are reversed, when calcium quantity drops under 0.74% (**Figure 5**, limit not shown).

**Figure 6:**
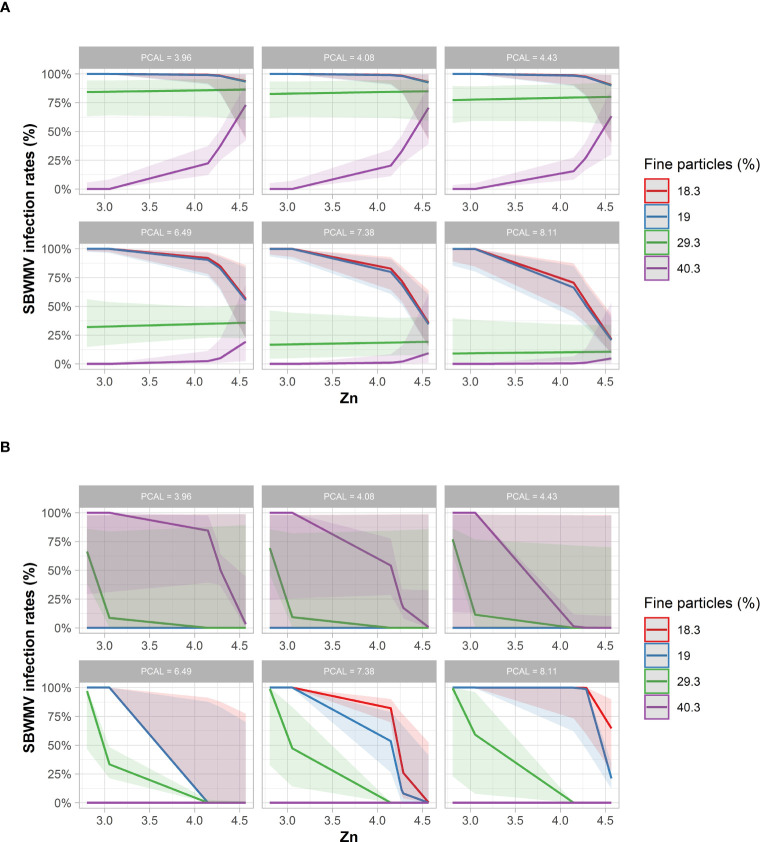
Predicted SBWMV infection rates based on soil parameters identified through STBM **(A)** and ST-MA **(B)**. Only the significant parameters for each model are presented. The colored zones depict 95% confidence intervals. PCAL: Calcium acetate lactate extractable phosphate (mg*100g^-1^ dry soil), Zn: plant-available zinc (mg*kg^1^ dry soil).

With respect to SBWMV infection rates, STBM predicted increased infection rates for soils with fine particle content of 29.3 mg*100g^-1^ dry soil and 40.3 mg*100g^-1^ dry soil (**Figure 6A**). For these high soil fine particle contents, a higher zinc content was predicted to further increase infection rates. For soils with a fine particle content of 18.4% mg*100g^-1^ dry soil and 19% mg*100g^-1^ dry soil, infection rates were predicted to decrease at a zinc content above 4.2 mg*kg dry soil^-1^ when the PCAL values were 4.43 mg*100g^-1^ dry soil or lower. At higher PCAL levels, the decrease in infection rates was more prominent and occurred already at lower since content.

**Figure 7:**
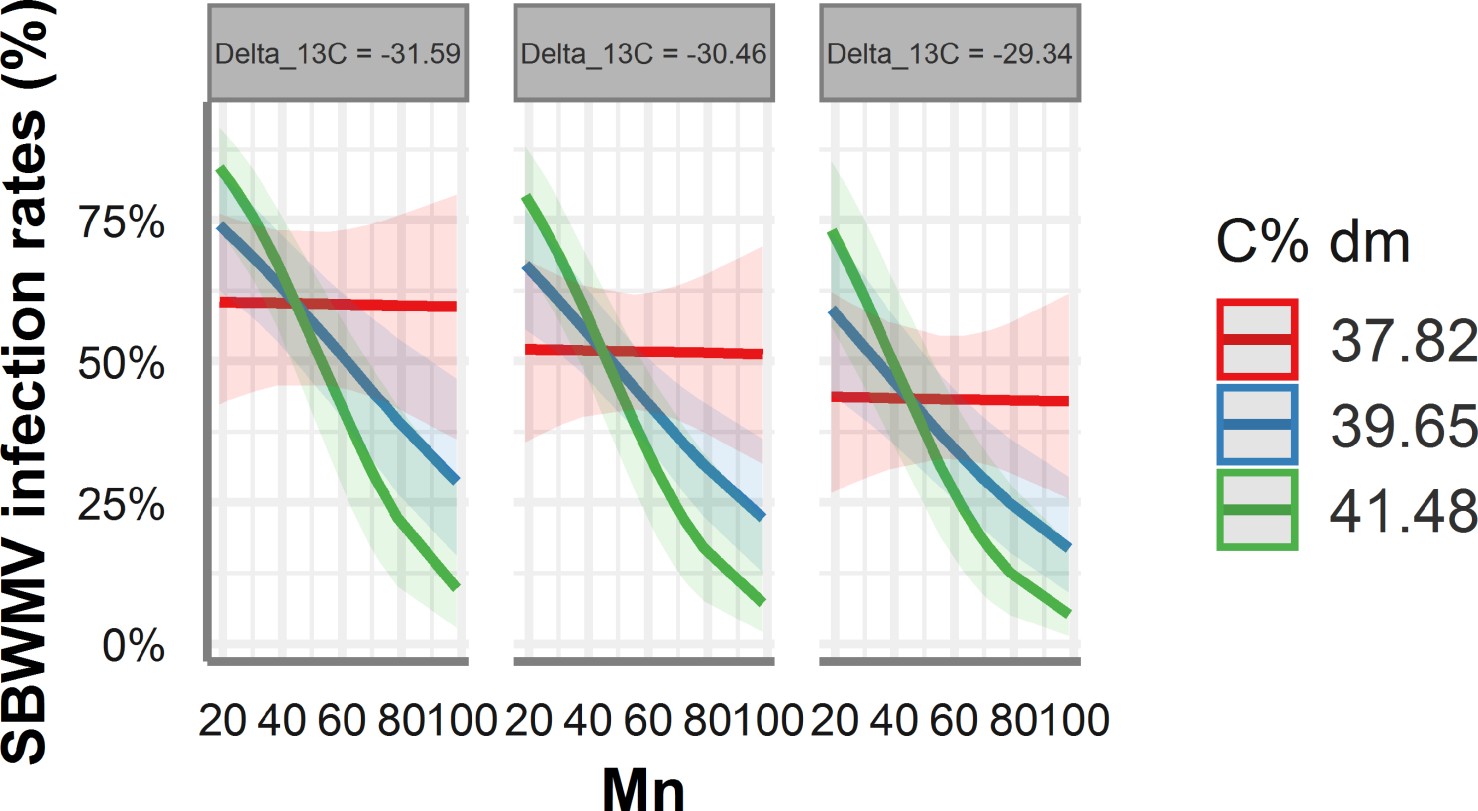
Predicted SBWMV infection rates based on modeling of element data measured in leaves. Only the significant parameters for the model are presented and only the four most represented parameters are shown in the figure. The colored zones depict 95% confidence intervals. Delta_13C: ^13^C/^12^C isotope ratio in the leaves, C% dm: % carbon content in the leaves, Mn: manganese (ppm) content in the leaves.

According to ST-MA, drastically reduced (100%) SBWMV infection rates were predicted at high PCAL values for soils with a high fine particle content (**Figure 6B**). By contrast, soils with low percentage of fine particles, infection rates were predicted to be high at high PCAL and high zinc concentrations. When the STBM and ST-MA algorithms were applied to model the effect of elements measured in leaves on SBWMV infection rates, both predicted very similar effects for the same set of parameters. The two models predicted increased infection rates at carbon contents above 38% and between 20ppm and 40 ppm manganese, and reduced infection rates at higher manganese concentrations (**Figure 7**). At low levels of carbon (38%), SBWMV infection rates were not influenced by manganese content. Furthermore, SBWMV infection rates increased at lower δ^13^C levels (**Figure 7**). None of the models achieved to predict SBWMV infection rates using the samples derived from roots.

### Analysis of the adequacy of the predictive model with respect to the observed data

**Figure 8:**
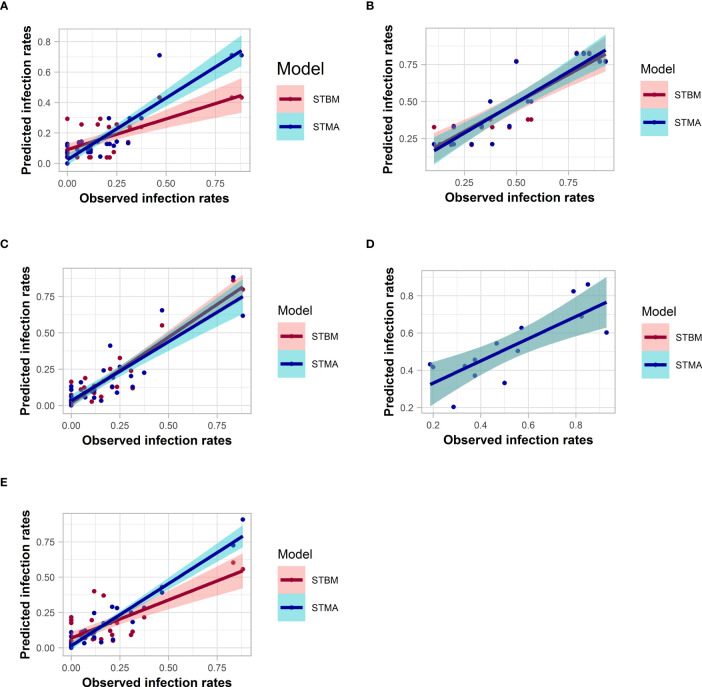
Correlation between the observed and predicted infection rates for SBMCV using soil **(A)**, leaf **(C)** or root **(E)** parameters, and for SBWMV using soil **(B)** and leaf **(D)** parameters. The colored zones depict 95% confidence intervals.

Most of the models fitted the data with an R² ranging from 0.47 to 0.89 for STBM and from 0.60 to 0.91 for ST-MA when plotting their respective predicted infection rates against the observations (**Figure 8** and **Table S5**). ST-MA performed better than STBM to predict infection rates of both SBCMV and SBWMV using soil parameters. ST-MA was better compared to STBM to predict SBCMV infection rates in root samples and SBWMV infection rates in leaf samples. ST-MA was calculated to have 3% (SBWMV, leaves) and 17% (SBWMV, roots) probability to lose information compared to 0.1% (SBCMV, leaves) and 27% (SBCMV, roots) respectively. By contrast, STBM performed better to predict SBCMV infection rates in leaf samples based on the measured parameters (with less than 0.1% probability to lose information by choosing ST-MA instead of STBM).

## Discussion

### Intrinsic soil parameters differentiate between the different origins of the soil and virus species present

In our study, we unraveled soil-related environmental factors influencing furovirus infection. We reasoned that soil, as it represents the support for *P. graminis* survival and mobility as well as the source for plant nutrition (Rao and Brakke, 1969; Kanyuka et al., 2003) may represent a key factor to influence furovirus epidemiology. We found that the interplay between soil nutrients, pH and soil structure resulted in a clear differentiation between the different soils as shown by PCA. Surprisingly, hierarchical ascendance clustering revealed a clear separation between SBWMV- and SBCMV infested soils, suggesting that each virus requires specific and distinct soil conditions for efficient infection, and that the measured parameters reflect these conditions. However, it is likely that the measured parameters are coupled to additional parameters not measured in this study, such as soil organic matter (Dequiedt et al., 2011), microbiome (Coller et al., 2019) or micronutrient composition (Hengl et al., 2017). As the availability of nutrients determines the nutritional status of plants (Holford and Mattingly, 1976; Sparks, 1980; Calabrese et al., 2017; Weih et al., 2018; Calabrese et al., 2019) it is important to also examine the differences in nutrients absorbed by plants grown in the different locations.

### The specific soil parameters that are predicted to influence furovirus infection rates in wheat presumably modulate *P. graminis* infection

Our study led to the identification of several soil factors influencing furovirus infection rates. Viruses, as obligate intracellular pathogens are unlikely to be directly affected by modulated soil parameters, in contrast to *P. graminis*, which lives in the soil. Therefore, furovirus infection rates probably represent a readout for *P. graminis* infection efficiency. However, a direct influence of the identified parameters on virus infection cannot be excluded.

The content of fine particles and plant available phosphorus (PCAL) was identified as parameters influencing infection rates of both virus species studied. In particular, ST-MA established that a decrease in soil fine particle quantity, leading to an increase of soil permeability (Alyamani and Şen, 1993) and an increase in plant available phosphorous increased SBWMV and SBCMV infection rates. These observations suggest that infection is promoted by permeable soils, facilitating the swimming of *P. graminis* zoospores to reach target root cells. Our results are consistent with previous studies, which used sand to multiply *P. graminis* (Adams and Swaby, 1988) or identified furovirus infection mostly in sandy soils (Kastirr et al., 2018). With respect to PCAL, which represents only the plant available part of the total phosphorous present in soils, a positive correlation was established between this parameter and furovirus infection rates. Interestingly, also amoeba abundance has been shown to increase in soils with increasing amounts of available phosphorous (Olatunji et al., 2018), supporting the idea that the germination of *P. graminis* zoospores from resting spores may be induced by the amount of available phosphorous. Consistently, it has been shown that root exudates as well as nutrient solution stimulate *Spongosphora subterranea* zoospore germination (Balendres et al., 2016; Balendres et al., 2018; Amponsah et al., 2021). Hence, increased plant-available P or increased soil fertility may lead to increased root exudation and consequently increased zoospore germination. Whether this would be for the benefit of infection or to its disadvantage may depend on other parameters like soil permeability and pH, which we indeed identified as parameters influencing infection rates in our models. With respect to pH, it is known that an increase in soil pH inhibits spore germination, a fact used in liming practices for agricultural management (Hwang et al., 2014; Amponsah et al., 2021). However, we only found an influence of pH on SBCMV infection rates in our experiments and our natural soil conditions only ranged from slightly acid to slightly alkaline. A slightly alkaline pH increased infection rates according to our models. Thus, factors coming along with a more alkaline pH, such as increased nitrification, may explain the increased infection rates at increased pH. Along with the before mentioned factors, an influence of Zn for SBWMV infection rates and Mg for SBCMV infection rates was detected. The influence of Zn on infection rates depended on the other parameters, i.e. fine particles and PCAL. Zn ions were shown to rapidly immobilize *P. graminis* zoospores in solution or at concentrations above 10 µg ml^-1^ (Adams and Swaby, 1988). For SBCMV, ST-MA predicted higher infection rates at higher Mg concentrations in the soil. A study on clubroot disease also identified magnesium as a factor influencing infection and clubbing (Myers and Campbell, 1985).

### Plant nutrient composition allows differentiation between SBCMV and SBWMV-infected soils and the prediction of parameters influencing infection rates

When PCA was performed with the measured plant parameters, the separation of the dataset into SBWMV and SBCMV-infected soils still occurred when PC2 was plotted vs. PC4 (**Figure 1C**). The highest variation in the dataset was explained by PC1 and related to the separation of the samples taken from leaves and roots, respectively. SBWMV and SBCMV are closely related viruses, sharing between 75% and 80% of homology on both RNAs (King et al., 2011) and are transmitted by same vector species. The fact that plant parameters can lead to the discrimination of SBWMV and SBCMV-infected soils suggests that either the difference in soil parameters selecting for SBWMV or SBCMV is reflected at the plant level or that the plant nutritional status, which is dependent on the soil parameters, supports infection by one or the other virus, respectively. We did not find significant differences in *P. graminis* species present in the roots of plants grown in SBWMV and SBCMV infected soils. This further confirms that the identified effects *in planta* are unlikely triggered or favored by *P. graminis* which is the common vector of both viruses.

Our models revealed common factors, which significantly influenced SBCMV infection rates in leaves and roots. It is important to stress, that we used natural field soils, thus the parameters we identify are parameters influencing infection under natural conditions. In contrast, many studies focusing on the influence of minerals on pathogen infection were conducted either with cultivated pathogens or by supplementing the tested nutrients, possibly in concentrations differing from *in vivo* situations (e.g. Holford and Mattingly, 1976; Aulakh and Dev, 1978; Adams and Swaby, 1988; Shevchenko et al., 2004; Soliman et al., 2020). In our study, one of the parameters identified to negatively influence infection rates was the increase of δ^15^N. Moreover, ST-MA predicted similar effects of the interaction between sulfur and calcium contents and of boron content in roots and leaves. The similarity of the observations obtained with two paired datasets (leaves and roots) confirms the robustness of the analysis.

Ratios of stable N-isotopes in plants reflect δ^15^N values in acquired N-sources (from soil and fertilizers) and the isotope discrimination in various metabolic processes (Craine et al., 2015). Losses of ^15^N depleted NH_3,_ N_2_, N_2_O and nitrate by volatilization, nitrification and denitrification in soils with high N-availability enrich the remaining soil pool by ^15^N (Szpak, 2014). Plants from soils fertilized with animal manures have higher δ^15^N (Choi et al., 2003; Szpak, 2014). Soil microbial biomass is ^15^N enriched and interaction with mycorrhiza accounts for variation in the isotope ratio (Hobbie and Högberg, 2012; Craine et al., 2015). Soil organic matter gets ^15^N enriched with age and extent of decomposition (Szpak, 2014). Soil microbial biomass and organic matter are again related to soil texture and pH (Dequiedt et al., 2011).

A higher δ^15^N value indicates conditions rich in organic nitrogen (Ariz et al., 2011; Craine et al., 2015), thus favorable for plant development. Nitrogen is also important for the biosynthesis of active plant defense compounds (Calabrese et al., 2017; Mur et al., 2017; Verly et al., 2020; Ding et al., 2021). Therefore, the predicted drop of SBCMV infection rates at high δ^15^N values could result, e.g. from interactions with soil microbiota or soil organic matter detrimental to the vector *Polymyxa graminis* or from increased plant fitness and capacity for active defense. A depletion of shoot and root δ^15^N related to pathogens has been described for viruses and nematodes in *Petunia* by Neilson et al. (1999). They consider natural abundances of stable isotopes as subtle integrators of whole-plant physiology and attribute changes in δ^15^N to metabolic reactions to the pathogens.

The increase of SBCMV infection rates with increasing boron quantities in leaves is consistent with studies showing increased boron quantities in virus infected plants (Overholt et al., 2009; Adkins et al., 2013), which may suggest that increased boron levels upon virus infection are a consequence of viral infection rather than a causality. However, boron is toxic at high concentrations, (e.g.(Camacho-Cristóbal et al., 2008; Hua et al., 2021). Boron toxicity could stress the plants and consequently weaken immunity to SBCMV infection. However, boron has also been described to limit virus symptoms in grapevine plants (Buoso et al., 2020). In the roots, the effect of boron differed dependent on calcium and sulfur levels. Sulfur is known to play an important role in plant defense as element in signaling- or defense molecules (Künstler et al., 2020; Zenda et al., 2021). Consistent with our data predicting an interaction of sulfur and calcium levels on SBCMV infection in leaves and roots, a recent study investigating the role of sulfur and calcium in plant fitness describes a role of both nutrients on photosynthetic performance and nitrogen metabolism in Indian mustard (Singh et al., 2018). Our study predicts a reduction of SBCMV infection rates in roots at increasing sulfur and calcium levels, but an opposite effect when only one reaches a high concentration. Further studies will be needed to understand the link between both of these elements and SBCMV infections.

The decrease of SBCMV infection rates at increasing copper concentrations observed in root samples may be explained by the anti-microbial and anti-viral properties of this element (Shevchenko et al., 2004; Soliman et al., 2020). We were not able to model the influence of nutrients and plant nutritional state on SBWMV infection rates in roots, due to small root sample numbers. For leaf samples, STBM and ST-MA identified the interaction between δ^13^C and manganese content to influence SBWMV infection rates.

Photosynthetic processes in C3 plants like wheat discriminate strongly against ^13^C (Farquhar et al., 1989). Stomatal closure, which occurs as a consequence e.g. of drought stress, by reducing CO_2_ availability (partial pressure within the leaf), reduces this discrimination and leads to increased (less negative) δ^13^C values. Nitrogen availability has the opposite effect and increases discrimination against ^13^C (Shangguan et al., 2000). Neilson et al. (1999) reported minor effects of nematodes and viruses on δ^13^C and limited to roots.

A decrease in δ^13^C may also be linked to a decrease in chlorophyll content (Soundararajan, 2012) or to an increase of secondary metabolites, such as defense compounds (Gleixner et al., 1993).

This may reflect a plant response to furovirus infection, especially as infected, symptomatic leaves display chlorotic stripes. The increase of the manganese content led to a drop of SBWMV infection rates. Previous studies indicated a similar effect of manganese, leading to an increase of resistance to plant disease (Huber and Haneklaus, 2007; Luo et al., 2020).

### Comparison of the model performance

Most of the models generated using the MAVG approach were very under dispersed, illustrating the importance of removing the non-significant factors from the model. Although ST-MA performed statistically better than STBM (except for the prediction of SBCMV infection rates using the data obtained from analysis of nutrients in leaves), it increases the complexity of the predictive model for a small gain of precision, only. This raises the question of the weight to give to the precision of the analysis in relation to the time and the cost of the extra analysis to reach it. However, during the analysis process, ST-MA is much faster compared to STBM, which requires to test every single possibility and factor one by one.

## Conclusion

Our study used soils obtained from different geographic locations to identify soil parameters and plant nutrients impacting SBCMV and/or SBWMV infection rates. The soil parameters probably influence *P. graminis* movement or generation of zoospores. Future experiments will test if the identified parameters can confirm the predictions of our model. Interestingly, our study suggests that SBCMV and SBWMV require specific soil conditions for infection, as our analysis of the soil parameters clearly separated soils containing SBCMV from soils containing SBWMV. This is specifically interesting with respect to the absence of fields reported to contain both furovirus species, despite they are both transmitted by *P. graminis* and, at least in Germany, co-occur in the same regions. The dataset containing plant samples grown in the different soils, was also separable into soils containing SBWMV and SBCMV, suggesting that the specific conditions required by SBWMV and SBCMV are transduced to the plant level. Finally, our study identified several plant parameters influencing virus infection. Future studies will test the effect of the identified factors on virus infection and may reveal, whether they represent the cause or rather the consequence of successful infection. This study contributes new insight into conditions determining furovirus infection in wheat and may help to develop strategies for nutrient-based pathogen management.

## Data availability statement

The original contributions presented in the study are included in the article/**Supplementary Material**. Further inquiries can be directed to the corresponding author.

## Author contributions

Conceptualization: AN and DPe; data curation and formal analysis: KG; funding acquisition: AN, DPe and FO; investigation: KG, DPa, MN, MH, CG; Methodology: KG, AN, DPa, MN, MH, CG, DPe, FO; Project administration: KG, DPa; DPe, AN; Supervision: AN, DPe; visualization: KG and AN; writing - original draft preparation: KG and AN; writing - review and editing: KG, DPa, MN, MH, CG, DPe, AN and FO. All authors contributed to the article and approved the submitted version.
