# Supplementary material for: Nutrients and soil structure influence furovirus infection of wheat
Source: Front Plant Sci. 2023 Aug 4;14:1200674. doi: 10.3389/fpls.2023.1200674 (PMC10436314; doi:10.3389/fpls.2023.1200674)
Supplement: Supplementary file 1 [file DataSheet_1.pdf]

Supplementary Material

Nutrients and soil structure influence furovirus infection of wheat

Kevin Gauthier[1], Dejana Pankovic[2], Miroslav Nikolic[3], Mirko Hobert[4], Christoph U. Germeier[5], Frank Ordon[2], Dragan Perovic[2], Annette Niehl[1*]

*** Correspondence:** Corresponding Author: Annette.niehl@julius-kuehn.de

1 Supplementary Figures and Tables

1.1 Supplementary Tables

Table S1. Location of fields from which the soil samples used in this study were derived. The viruses present in these field were identified by Kastirr and colleagues (Kastirr *et al.*, 2018)

Field location	Abbreviation	Cultural season sampling	Virus present
Bologna (Italy)	Bol_17	2017	SBCMV
	Bol_18	2018	
	Bol_19	2019	
	Bol_20	2020	
Elxleben (Germany)	Elxl_18	2018	SBWMV
	Elxl_19	2019	
	Elxl_20	2020	
Heddesheim (Germany)	Hed_19	2019	SBWMV
pure Kent (UK)	Ktp_18	2018	SBCMV
used Kent (UK)	Ktg_18	2018	SBCMV
Vatan (France)	Vat_18	2018	SBCMV

	Vat_19	2019	
	Vat_20	2020	
Westerrade (Germany)	Wes_18	2018	SBWMV
	Wes_19	2019	
pure Wiltshire (UK)	Wlp_18	2019	SBCMV
used Wiltshire (UK)	Wlg_19	2019	SBCMV

Table S2: Primers used to amplify *P. graminis* and SBWMV RNA

**Primer name**	**Direction**	**Role**	**Sequence**
PGG_51_FW	Forward	PCR (both subspecies)	GCTACAATGCTAGGTTCAACGAGTC
PGG_882_RV	Reverse	PCR (both subspecies)	CGCTTATTGATATGCTTAAATTCGG
PGG_166_FW	Forward	Nested PCR (both subspecies)	GGAATTCCTATAGACGCAGGTCATC
PGG_631_RV	Reverse	Nested PCR (both subspecies)	CTTGCGTTCAAAGATTCGATGATT
PTP_409_FW	Forward	RT-qPCR ssp. *tepida*	GTAAAAATGTGGATCGTCTCTGTTG
PTP_512_RV	Reverse	RT-qPCR ssp. *tepida*	CAATTCGACTTTAGCCACCGTT
PTM_403_FW	Forward	RT-qPCR ssp. *temperata*	GGATTGTGGGCTATGTGACC
PTM_481_RV	Reverse	RT-qPCR ssp. *temperata*	ATCTTATGATTCCATTAGCCAATTCTC
1-BWF-6237	Forward	PCR SBWMV	GTTGGGTTATGATAAAAGTCTGAAGATG
1-BWR-7018	Reverse	PCR SBWMV	CCTCATCATCGCTAAATGTTGATCT
1-BWF-6541T	Forward	RT-qPCR SBWMV	TTGGCTGAGGAAGGAAAGG
1-BWR-6683	Reverse	RT-qPCR SBWMV	GACCGAAAGGAATATATAGTAACACGTA AAC

Table S3: Excel sheet summarizing all physical and chemical soil and plant parameters measured in this study

Table S4A and B: Identification of the virus (A) and vector species (B) contained in plant roots grown in the different soils for 12 weeks. For both tables, the sample names are described by the following : Field name (Bol: Bologna, Elxl: Elxleben, Kt: new Kent, Kt_gbr: used Kent, Hdsm: Heddesheim, Vt: Vatan, Wes: Westerrade, Wil: new Wiltshire, Wil_gbr: used Wiltshire)_ year of sampling_cultivar name (AVA: Avalon, PES: durum Pescadou, PRE: Prevert). 4a: SBWMV_Tm: melting temperature of the amplicon generated during qRT-PCR with SBWMV-specific primers, SBWMV_Ct1 / SBWMV_Ct2: Threshold cycle number measured during qRT-PCR amplification with SBWMV-specific primers for the two technical replicates, ELISA Status: Results from ELISA (using antibodies recognizing both SBWMV and SBCMV) on single plants, qRT-PCR SBWMV: sanitary status obtained after qPCR analysis on SBWMV RNA1 on root pools. +: at least one plant (ELISA) or the pool (qPCR) is positive, -: all plants (ELISA) or the pool (qPCR) is negative. 4b: TMP_Tm: melting temperature of the amplicon generated during qRT-PCR with *P. graminis ssp. temperata*-specific primers, TMP_Ct1 / TMP_Ct2: Threshold cycle number measured during qRT-PCR amplification with *P.graminis ssp. temperata*-specific primers for two technical replicates, TMP Status: Sanitary status of the tested pools regarding *P. graminis ssp. temperata* with +: infected, -: uninfected, TPD_Tm: melting temperature of amplicon generated during qRT-PCR with *P.graminis ssp. tepida*-specific primers, TPD_Ct1 / TPD_Ct2: Threshold cycle number measured during qRT-PCR amplification with *P.graminis ssp. tepida*-specific primers for the two technical replicates, TPD Status: Sanitary status of the tested pools regarding *P. graminis ssp. tepida* with +: infected, -: uninfected. Elxleben plants from the year 2020 were analyzed separately and were positive in ELISA and in qRT-PCR for SBWMV, both ssp. of *P. graminis* were detected in the roots of each cultivar (data not shown).

A

Sample	SBWMV_Tm	SBWMV_Ct1	SBWMV_Ct2	ELISA Status	qRT-PCR SBWMV
Positive Control	78,4	9,02	8,79	+	+
BOL_18_AVA	62,8	40	40	+	-
BOL_18_PES	60,7	40	40	+	-
BOL_18_PRE	60,85	40	40	-	-
BOL_17_AVA	61,6	40	40	+	-
BOL_17_PES	69,35	40	34,5	+	-

BOL_17_PRE	68,95	33,59	40	+	-
BOL_20_AVA	60,85	40	40	+	-
BOL_20_PES	62,3	40	40	+	-
BOL_20_PRE	60,65	40	40	-	-
BOL_19_AVA	61,6	40	40	+	-
BOL_19_PES	62,65	40	40	+	-
BOL_19_PRE	61,35	40	40	-	-
ELXL_18_AVA	60,2	40	40	+	-
ELXL_18_PES	78,25	31,86	0	+	+
ELXL_18_PRE	78,2	32,27	0	+	+
KT_GBR_18_AVA	62,3	40	40	+	-
KT_GBR_18_PES	61,25	40	40	+	-
KT_GBR_18_PRE	60	40	40	+	-
HDSM_19_AVA	77	33,58	31,95	+	+
HDSM_19_PES	77	29,09	28,67	+	+
HDSM_19_PRE	76,95	29,52	29,48	+	+
VT_18_AVA	66	40	36,96	-	-
VT_18_PES	60,35	40	40	+	-
VT_18_PRE	62,45	40	40	-	-
VT_20_AVA	61	40	40	+	-
VT_20_PES	60,35	40	40	+	-
VT_20_PRE	62,65	40	40	+	-
WES_19_AVA	75,1	34,82	40	+	-

WES_19_PES	77	40	33,55	+	+
WES_19_PRE	77,15	31,99	33,08	+	+
WIL_GBR_18_AVA	67,25	40	35,63	+	-
WIL_GBR_18_PES	63,6	40	40	+	-
WIL_GBR_18_PRE	62,75	40	40	+	-
WES_18_AVA	77,25	30,92	32,43	+	+
WES_18_PES	61,2	40	40	+	-
WES_18_PRE	77,05	31,64	33,15	+	+
VT_19_AVA	68,6	35,5	40	+	-
VT_19_PES	61,5	40	40	+	-
VT_19_PRE	64,25	40	40	-	-
ELXL_19_AVA	63,05	40	40	+	-
ELXL_19_PES	60,55	40	40	+	-
ELXL_19_PRE	67,25	39,34	40	+	-
KT_18_AVA	62,8	40	40	-	-
KT_18_PES	61,25	40	40	-	-
KT_18_PRE	63,45	40	40	-	-
WIL_18_AVA	64,45	40	40	-	-
WIL_18_PES	61,7	40	40	-	-
WIL_18_PRE	63,85	40	40	-	-

B

Sample	TMP_Tm	TMP_Ct1	TMP_Ct2	TMP Status	TPD_Tm	TPD_Ct1	TPD_Ct2	TPD Statut

Positive Control	79,5	19,27	18,45	+	81,22	10,59	11,15	+
BOL_18_AVA	62,05	39,54	40	-	81,15	29,54	29,38	+
BOL_18_PES	63,4	40	40	-	61,7	40	40	-
BOL_18_PRE	67,9	31,86	40	-	60,65	40	40	-
BOL_17_AVA	62,35	36,69	40	-	81,05	29,89	29,01	+
BOL_17_PES	67,95	33,32	40	-	60,45	40	40	-
BOL_17_PRE	67,4	33,13	40	-	81	28,02	27,26	+
BOL_20_AVA	67	40	36,6	-	60,55	40	40	-
BOL_20_PES	60	40	40	-	60,9	40	40	-
BOL_20_PRE	61,15	40	40	-	81,2	27,29	26,91	+
BOL_19_AVA	67,85	33,11	40	-	81,05	28,55	28,46	+
BOL_19_PES	61,6	40	40	-	81,05	27,68	28,31	+
BOL_19_PRE	62,2	40	40	-	81,05	29,4	31,61	+
ELXL_18_AVA	61	40	40	-	60	40	40	-
ELXL_18_PES	60	40	40	-	81,25	27,33	27,68	+
ELXL_18_PRE	62,7	40	40	-	81,3	26,15	26,22	+
KT_GBR_18_AVA	60,7	40	40	-	81,35	23,25	23,36	+
KT_GBR_18_PES	61,15	40	40	-	81,2	24,37	24,25	+
KT_GBR_18_PRE	60,65	40	40	-	81,35	24,57	24,44	+
HDSM_19_AVA	63,65	40	40	-	81,5	25,22	25,22	+
HDSM_19_PES	67,6	33,59	40	-	81,4	27,97	28,5	+
HDSM_19_PRE	60	40	40	-	81,4	25,81	25,32	+
VT_18_AVA	60,5	40	40	-	81,3	24,04	24,08	+

VT_18_PES	60,45	40	40	-	81,2	27,33	26,88	+
VT_18_PRE	64,45	40	40	-	60,9	40	40	-
VT_20_AVA	79,65	26,2	26,58	+	81,5	26,1	26,47	+
VT_20_PES	73,25	33,93	33,64	-	81,5	26,87	26,9	+
VT_20_PRE	70,55	40	32,23	-	81,3	28,27	28,16	+
WES_19_AVA	61,2	40	40	-	81,15	26,7	26,33	+
WES_19_PES	61,55	40	40	-	81,15	25,19	25,23	+
WES_19_PRE	61,8	40	40	-	71,45	40	30,51	-
WIL_GBR_18_AVA	60	40	40	-	81,65	20,68	20,6	+
WIL_GBR_18_PES	70	40	37,25	-	81,4	22,62	22,43	+
WIL_GBR_18_PRE	61,4	40	40	-	81,45	24,88	25	+
WES_18_AVA	72,15	33,79	40	-	70,55	40	40	-
WES_18_PES	73,45	34,18	35,33	-	61,5	40	40	-
WES_18_PRE	63,4	40	40	-	61,85	40	40	-
VT_19_AVA	61,5	40	40	-	81,35	23,13	23,41	+
VT_19_PES	67,3	40	34,43	-	81,45	26,03	25,99	+
VT_19_PRE	62,2	40	40	-	71,8	40	32,11	-
ELXL_19_AVA	60	40	40	-	61,5	40	40	-
ELXL_19_PES	78,7	33,75	0	+	64,2	40	0	-
ELXL_19_PRE	66,65	40	33,13	-	61	40	40	-
KT_18_AVA	72,25	33,25	40	-	81,5	23,72	23,76	+
KT_18_PES	78,9	33,35	33,33	+	81,55	24,78	24,96	+
KT_18_PRE	79,95	30,65	30,05	+	81,35	25,13	27,89	+

WIL_18_AVA	66	40	40	-	81,4	27,48	27,6	+
WIL_18_PES	60,35	40	40	-	81,35	26,25	25,72	+
WIL_18_PRE	63,7	40	40	-	61,7	40	40	-

Table S5: Comparison between observed infection rates and predictions provided by STBM and STMA modelling. sg: significance, Adj. R2: adjusted R-square, AIC: Akaike information criterion. Observed: Infection rates measured by ELISA. The best modeling parameters for each set are displayed in bold.

Model	STBM					STMA				
	Value	Error	sg	Adj. R2	AIC	Value	Error	sg	Adj. R2	AIC
				Soil SBCMV						
Intercept	0.091	0.020	7-71E^{-5}	-	-	**0.024**	**0.018**	**1.9E^{-1}**	-	-
Observed	0.403	0.074	5.78E^{-6}	0.474	-60.7	**0.813**	**0.067**	**2.6E^{-13}**	**0.823**	**-67.6**
				Leaves SBCMV						
Intercept	**0.018**	**0.015**	**2.40E^{-1}**	**-**	**-**	0.032	0.021	1.36E^{-1}	-	-
Observed	**0.904**	**0.056**	**1.61E^{-16}**	**0.890**	**-74.4**	0.813	0.078	1.13E^{-11}	0.771	-57.2
				Roots SBCMV						
Intercept	0.069	0.022	3.69E^{-3}	-	-	**0.015**	**0.014**	**2,95E^{-1}**	**-**	**-**
Observed	0.540	0.081	1.96E^{-7}	0.587	-53.4	**0.879**	**0.051**	**4.02E^{-17}**	**0.91**	**-82.7**
				Soil SBWMV						
Intercept	0.105	0.057	8.54E^{-2}	-	-	**0.079**	**0.052**	**1.45E^{-1}**	**-**	**-**
Observed	0.770	0.105	1.58E^{-6}	0.758	-21.3	**0.828**	**0.095**	**1.73E^{-7}**	**0.816**	**-24.8**

Leaves SBWMV										
Intercept	**0.212**	**0.075**	**$1.52E^{-2}$**	**-**	**-**	**0.212**	**0.075**	**$1.52E^{-2}$**	**-**	**-**
Observed	**0.6**	**0.132**	**$6.90E^{-4}$**	**0.600**	**-16.5**	**0.6**	**0.132**	**$6.90E^{-4}$**	**0.600**	**-16.5**
Roots SBWMV										
Intercept	-	-	-	-	-	-	-	-	-	-
Observed	-	-	-	-	-	-	-	-	-	-

1.2 Supplementary Figures

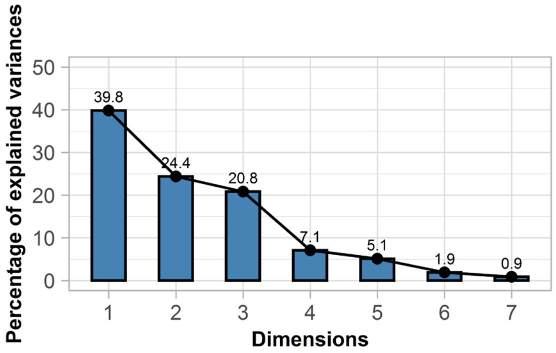

Figure S1: Eigenvalues per variance of each principal component for the soil analysis. The numbers indicate the proportion of information retained by each dimension.

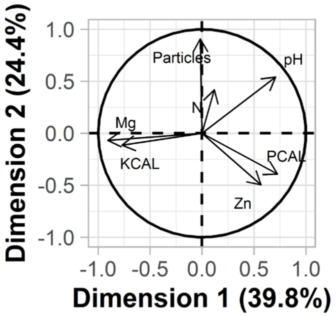

Figure S2: Coordinate circle of the PCA based on soil properties. Data were normalized prior to the analysis. Normalization was by subtracting the average value for a given parameter from the value of the respective parameter and division by the standard deviation. Particles: percentage of fine particles in the soil, N: nitrogen content (%), PCAL: calcium acetate lactate extractable phosphate (mg*100mg^{-1} dry soil), Zn: zinc content (mg*kg^{-1} dry soil), KCAL: calcium acetate lactate extractable potassium (mg*100mg^{-1} dry soil), Mg: magnesium content (mg*100mg^{-1} dry soil).

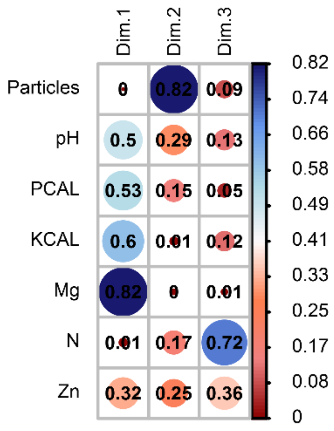

Figure S3: Square cosine, squared coordinates of the PCA performed on soil parameters. Data were normalized prior to the analysis. Dim.1, Dim.2, Dim.3 indicate the different dimensions of the dataset. Particles: percentage of fine particles in the soil, N: nitrogen content (%), PCAL: calcium acetate lactate extractable phosphate (mg*100mg^{-1} dry soil), Zn: zinc content (mg*kg^{-1} dry soil), KCAL: calcium acetate lactate extractable potassium (mg*100mg^{-1} dry soil), Mg: magnesium content (mg*100mg^{-1} dry soil).

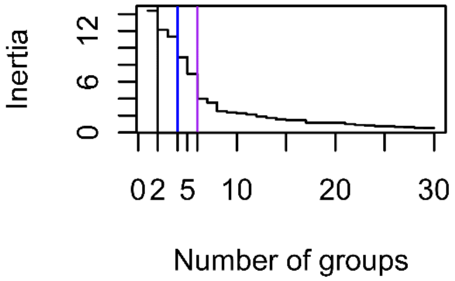

Figure S4: Drop of inertia among the hierarchical classification. The highest drop occurs for six and then four classes represented by purple and blue dashed lines, respectively.

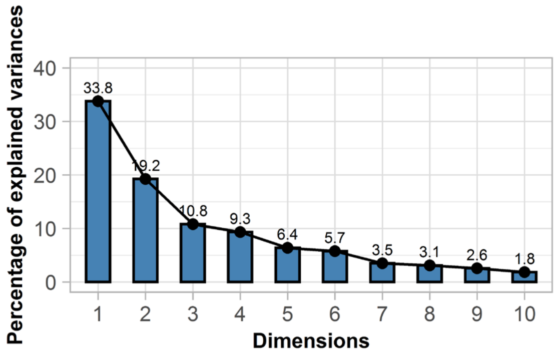

Figure S5: Eigenvalues per variance of each principal component for the analysis of plant samples. The numbers indicate the proportion of information retained by each dimension.

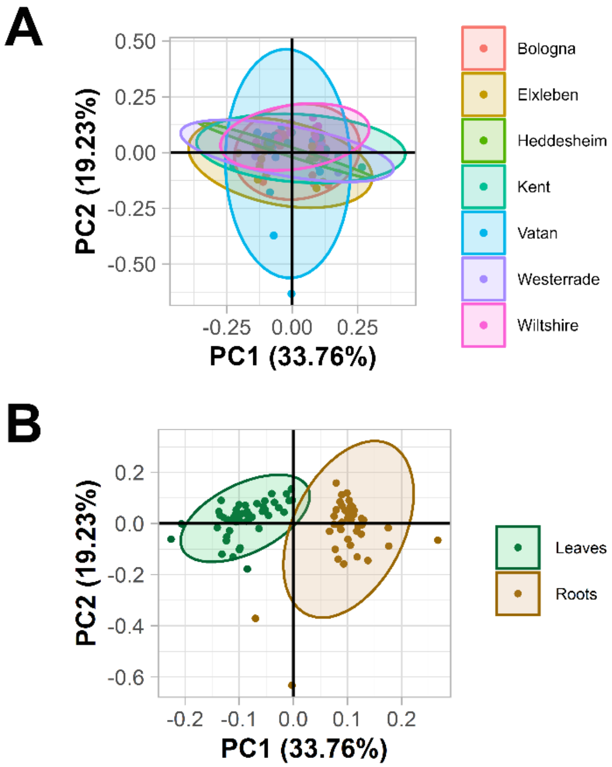

Figure S6: Principal component analysis of leaf and root samples of plants grown in viruliferous P. graminis-containing soil. Samples were taken from plants grown in different field soils, respectively for twelve weeks. Thirteen element parameters were analyzed after normalization. 95% confidence intervals are represented by ellipses of different colors, representing the field of origin (A) or the plant roots or foliage, respectively (B).

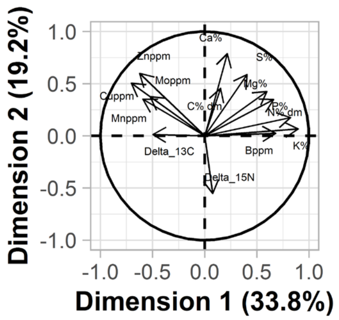

Figure S7: Coordinate circle of the PCA on root and leaf samples. Data were normalized prior to the analysis. The abbreviations represent the respective quantities of: carbon (C% dm, in % of the dry mass), nitrogen (N% dm, in % of the dry mass), boron (Bppm, in ppm), calcium (Ca, %,), copper (Cu, ppm), potassium (K, %), magnesium (Mg, %), manganese (Mn, ppm), molybdenum (Mo,ppm),

phosphorous (P, %), sulfur (S, %), zinc (Zn, ppm), δ13C (Delta_13C, ‰ deviation from belemnite of the pee dee formation), δ15N (Delta_15N, ‰ deviation from the standard air).

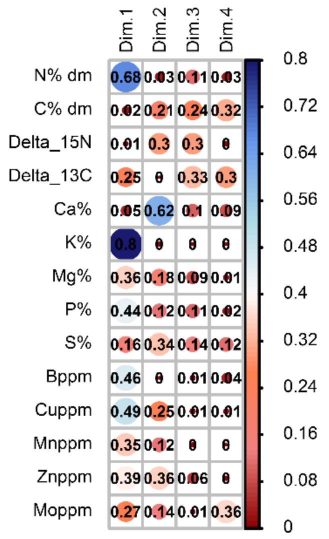

Figure S8: Square cosine, squared coordinates of the PCA performed on root and leaf samples. Data were normalized prior to the analysis. The abbreviations represent the respective quantities of: : carbon (C% dm, in % of the dry mass), nitrogen (N% dm, in % of the dry mass), boron (Bppm, in ppm), calcium (Ca, %,), copper (Cu, ppm), potassium (K, %), magnesium (Mg, %), manganese (Mn, ppm), molybdenum (Mo,ppm), phosphorous (P, %), sulfur (S, %), zinc (Zn, ppm), δ13C (Delta_13C, ‰ deviation from belemnite of the pee dee formation), δ15N (Delta_15N, ‰ deviation from the standard air).
